# Identification and characterization of a novel QTL for barley yellow mosaic disease resistance from bulbous barley

**DOI:** 10.1002/tpg2.20557

**Published:** 2025-01-13

**Authors:** Yi Hong, Hui Zhou, Mengna Zhang, Yuhang Zhang, Juan Zhu, Chao Lv, Baojian Guo, Feifei Wang, Qingliang Li, Jie Sun, Rugen Xu

**Affiliations:** ^1^ Key Laboratory of Plant Functional Genomics of the Ministry of Education/Jiangsu Key Laboratory of Crop Genomics and Molecular Breeding/Jiangsu Co‐Innovation Center for Modern Production Technology of Grain Crops/Joint International Research Laboratory of Agriculture and Agri‐Product Safety of Ministry of Education of China Yangzhou University Yangzhou China; ^2^ China Resources Beer (Holdings) Company Limited Beijing China

## Abstract

Winter barley (*Hordeum vulgare*) production areas in the middle and lower reaches of the Yangtze River are severely threatened by barley yellow mosaic disease, which is caused by *Barley yellow mosaic virus* and *Barley mild mosaic virus*. Improving barley disease resistance in breeding programs requires knowledge of genetic loci in germplasm resources. In this study, bulked segregant analysis (BSA) identified a novel major quantitative trait loci (QTL) *QRym.ZN1‐7H* for barley yellow mosaic disease resistance in an F_2_ population derived from the cross between “Nongke 1–6” (*H. vulgare*) and “Zaoshu 3” (*H. vulgare*). This QTL, originating from bulbous barley (*Hordeum bulbosum*), demonstrated stability and was further validated in another F_2_ population derived from the cross between “Nongke 2–6” (*H. vulgare*) and “Supi 1” (*H. vulgare*). *QRym.ZN1‐7H* accounted for 10.61%–19.34% of the phenotypic variance. The QTL was further fine mapped to the 14‐ to 39‐Mb interval on barley chromosome 7H. Transcriptome analysis identified 53 and 35 differentially expressed genes in roots and leaves (at *QRym.ZN1‐7H* locus), respectively, with nine genes differentially expressing in both tissues. *HORVU.MOREX.r3.7HG0650990*, a member of the disease resistance protein family (NBS‐LRR class), is the most likely candidate gene for *QRym.ZN1‐7H*. Enrichment analysis indicated that *QRym.ZN1‐7H* may be involved in signal transduction in plant innate immune response. This study laid a foundation for barley disease resistance breeding.

## Core Ideas

- A novel quantitative trait loci (QTL) (*QRym.ZN1‐7H*) for barley yellow mosaic disease resistance was identified from bulbous barley.
- *QRym.ZN1‐7H* was fine mapped to the 14‐ to 39‐Mb interval on chromosome 7H.
- Transcriptome analysis identified nine differentially expressed genes both in roots and leaves (at *QRym.ZN1‐7H*).
- *QRym.ZN1‐7H* may be involved in signal transduction in plant innate immune response.

**Abbreviations**
BaMMV: barley mild mosaic virus
BaYMV: barley yellow mosaic virus
BSA: bulked segregant analysis
BYMD: barley yellow mosaic disease
CTAB: cetyltrimethylammonium bromide
FPKM: fragments per kilobase of transcript per million fragments mapped
GO: Gene Ontology
KEGG: Kyoto Encyclopedia of Genes and Genomes
LOD: logarithm of the odds
PCR: polymerase chain reaction
QTL: quantitative trait loci
sAUDPS: standard area under the disease progress stairs
SLAF: specific‐locus amplified fragment sequencing
SNP: single‐nucleotide polymorphism

## 1 INTRODUCTION

Barley yellow mosaic disease (BYMD) is a soil‐borne virus disease caused by single or combined infection of *Barley yellow mosaic virus* (BaYMV) and *Barley mild mosaic virus* (BaMMV). Under natural conditions, these two viruses parasitize in the resting spores of *Polymyxa graminis* in the soil and use them as mediators to infect plant roots, thereby spreading the virus (J. Chen, 1992). Virus‐carrying spores of *P. graminis* germinate and infect roots of barley seedlings in autumn (Jiang et al., 2020; Kang et al., 2005) and further transmitted to leaves, especially new leaves, in the following spring, resulting in chlorotic spots during the vegetative growth period. When the ambient temperature exceeded 20°C, the chlorotic spots disappeared, thus showing the “cryptic disease” phenomenon. At the jointing stage, the susceptible plants exhibit stunted growth, with dwarfed plants and reduced tiller numbers. The infected plants also tend to mature late. BYMD can reduce barley yields by 10%–30%, and in severe cases, it can cause yields losses of 70%–80% or even result in total crop failure (Plumb et al., 1986).

Mining resistance genes and breeding disease‐resistant varieties are the most economical and effective way of controlling BYMD. Over 20 BYMD resistance loci/genes have been reported but only two genes, *eIF4E* and *PDIL5‐1*, have been cloned. Among these reported genes, *rym4* is derived from southern European landrace “Ragusa,” which is resistant to the European virus strains of BaMMV and BaYMV but susceptible to BaYMV‐2 (Kanyuka et al., 2005). *rym5* is derived from a Chinese landrace “Mushigang 3” (Stein et al., 2005) and shows resistance to BaMMV, BaYMV‐1, and BaYMV‐2 but is susceptible to the emerging BaMMV‐Teik. *rym6*, derived from the Japanese landrace “Amagi Nijo,” shows only resistance to the Japanese virus strain BaYMV‐II but is susceptible to all European BaMMV and BaYMV (Zhang et al., 2024). *rym10* is derived from the German variety “Hiberna” and confers resistance to the European virus strains of BaYMV and BaYMV‐2 (Kanyuka et al., 2005). *rym_HOR4224_*, derived from the Japanese landrace “HOR4224,” shows specific resistance to BaMMV and susceptible to BaYMV (Perovic et al., 2014). *rym_HOR3298_* derived from the Iranian local variety “HOR3298” shows broad‐spectrum resistance to multiple isolates of BaYMV/BaMMV (Shi et al., 2019). All above six genes are allelic variants derived from *eIF4E*, a gene on chromosome 3H that encodes eukaryotic translation initiation factor 4E. *Rym17* is also located on chromosome 3H, showing resistance to BaYMV‐I and BaYMV‐III (Kai et al., 2012).

*rym1* is immune to all European virus strains and Japanese virus strains BaYMV‐I, BaYMV‐II, BaMMV‐Nal, and BaMMV‐Kal, and moderately resistant to Japanese virus strain BaYMV‐III (Okada et al., 2003; Yang, Habekuß, et al., 2014). *rym11*, derived from the Russian landrace “Russia 57,” shows broad‐spectrum resistance to all European virus strains (Lüpken et al., 2013). Both *rym1* and *rym11* are allelic variants derived from *PDIL5‐1*, a member of the protein disulfide isomerase (PDI) gene family on chromosome 4H (Yang, Lüpken, et al., 2014). *rym8*, *rym9*, *rym12*, *rym13*, and *rym18* are all located on chromosome 4HL. Among them, *rym8* is resistant to BaYMV‐1 and BaMMV, which are widely distributed in Europe (Bauer et al., 1997). *rym9* was only resistant to BaMMV (Werner et al., 2005). *rym12* and *rym13* show resistance to all current BaYMV and BaMMV strains in Europe (Götz & Friedt, 1993; Humbroich et al., 2010; Werner et al., 2003). *rym18* showed resistance to Japanese virus strains BaYMV‐I and BaYMV‐III (Kai et al., 2012).

*rym7* is located near the centromere on chromosome 1H, resistant to the BaMMV‐Teik, partially resistant to BaMMV‐SIL and BaMMV, and susceptible to BaYMV and BaYMV‐2 (Yang et al., 2013). The bulbous barley, a wild relative of barley, carries *Rym14^Hb^* and *Rym16^Hb^* on chromosomes 2H and 6H, respectively, conferring resistance to BaYMV and BaMMV in barley (Pidon et al., 2021; Ruge et al., 2003; Ruge‐Wehling et al., 2006). *rym3* and an unnamed locus from “Chikurin Ibaraki 1” share the same genetic region on chromosome 5HS (Saeki et al., 1999; Werner et al., 2003). *rym3* is highly resistant to BaYMV‐I, BaYMV‐II, and BaYMV‐V but highly susceptible to BaMMV. The unnamed locus is resistant to BaYMV and BaYMV‐2 and susceptible to BaMMV. *rym15* is located on chromosome 6HS and is only resistant to BaMMV (Le Gouis et al., 2004). *rym2* and *rym7t* are in the same interval on chromosome 7HL, and *rym2* is resistant to BaMMV and BaYMV (Takahashi et al., 1973), while *rym7t* is resistant to BaYMV (Takata et al., 2012).

BaYMV and BaMMV are both single‐stranded RNA viruses. Their genomes can mutate rapidly and co‐evolve with resistance genes in resistant varieties, leading to a variety of pathogenic strains (Yang et al., 2017). Eventually, these pre‐existing resistance genes will lose their resistant function as the BYMD strains change. To ensure the safety of barley production, novel BYMD resistance loci/genes need to be explored and applied. *Rym14^Hb^* and *Rym16^Hb^* with broad‐spectrum resistance among the 22 barley BYMD resistance genes reported were identified from the bulbous barley. Interspecific hybridization between cultivated barley “Supi 1” susceptible to BYMD and disease‐resistant bulbous barley “GBC141” generated two disease‐resistant translocation lines known as “Nongke 1–6” and “Nongke 2–6” (Li et al., 1999). Previous studies have confirmed that these two translocation lines are immune to BYMD in Jiangsu disease nursery and carry novel genes resistant to Jiangsu virus strains (Pan et al., 2022). In this study, an F_2_ population derived from the cross between “Zaoshu 3” (a susceptible barley variety) and “Nongke 1–6” was used to identify the novel resistance quantitative trait loci (QTL) of BYMD resistance through bulk segregant analysis sequencing (BSA‐seq) analysis. The novel QTL was then verified using another F_2_ population derived from a cross between “Supi 1” (a susceptible barley variety) and “Nongke 2–6.” Near‐isogenic lines (NILs) differing in the target interval were used to fine map the gene on 7H. This study will facilitate the cloning of new resistance genes.

## 2 MATERIALS AND METHODS

### 2.1 Plant materials and field trials

BYMD susceptible varieties “Zaoshu 3” and “Supi 1” were utilized as female parents. Two disease‐resistant male parents, “Nongke 1–6” and “Nongke 2–6,” were derived from an interspecific hybrid between “Supi 1” and the BaYMV‐resistant *Hordeum bulbosum* accession “GBC141”. A previous study confirmed that both “Nongke 1–6” and “Nongke 2–6” contain genetic material from bulbous barley (Li et al., 1999). “Zaoshu 3” was crossed with “Nongke 1–6” to develop an F_2_ population (abbreviated as ZN1‐F_2_), which was employed for the detection of novel resistant QTL from bulbous barley. “Supi 1” was crossed with “Nongke 2–6” to generate another F_2_ population (abbreviated as SN2‐F_2_) for further verification of the identified QTL.

**Core Ideas**

- A novel quantitative trait loci (QTL) (*QRym.ZN1‐7H*) for barley yellow mosaic disease resistance was identified from bulbous barley.
- *QRym.ZN1‐7H* was fine mapped to the 14‐ to 39‐Mb interval on chromosome 7H.
- Transcriptome analysis identified nine differentially expressed genes both in roots and leaves (at *QRym.ZN1‐7H*).
- *QRym.ZN1‐7H* may be involved in signal transduction in plant innate immune response.

In the autumn of 2019, ZN1‐F_2_ (comprising 441 individuals) and the parents were sown in the BYMD nursery at Yangzhou University (Yangzhou, Jiangsu Province, China, 32° N, 119° E). In the autumn of 2020, SN2‐F_2_ (comprising 202 individuals) and the parents were sown in the same nursery. The F_2_ population and parents were planted in rows of 1.2 m in length, with each row containing 20 seeds and a row spacing of 0.2 m. The parental lines were sown in two rows, with three replications. An individual from the ZN1‐F_2_, exhibiting a heterozygous genotype within the candidate interval of *QRym.ZN1‐7D*, was self‐pollinated three times to generate NILs using the single‐seed descend method. In the F_2:5_ generation, a pair of near‐isogenic lines (ZN1‐6011^7D+^ and ZN1‐6012^7D−^) were then selected and subsequently planted in the diseased field to investigate differences in resistance to BYMD in the autumn of 2023. All field trial management adhered to standard procedures for barley cultivation.

### 2.2 Barley yellow mosaic disease assessment

The level of BYMD was scored on a scale of 0–4 in the nursery (Pan et al., 2021). Grade 0: no visible symptoms on the new leaves. Grade 1: chlorotic spots appear with less than 5% of new leaves turning chlorotic. Grade 2: chlorotic spots increase, and short striped spots parallel to the leaf veins appear, with 5%–25% of new leaves turning chlorotic. Grade 3: chlorotic spots expand significantly and develop into a mosaic‐like appearance, with a slight decrease in plant height and 25%–75% of new leaves turning chlorotic. Grade 4: barley leaves turn yellow; plants shrink and die, with more than 75% of new leaves turning chlorotic.

The standard area under the disease progress stairs (sAUDPS) index is commonly used for resistance evaluation of BYMD. It can fit multi‐stage disease grade data and is not limited by the number and duration of investigation and can more accurately assess the incidence degree of yellow mosaic disease (Simko & Piepho, 2012; Simko et al., 2015). The index is calculated as follows:

(1) $$\begin{array}{ccc} \mathrm{sAUDPS} & = & \left\{\sum_{i=1}^{n\;-1}\left[\frac{y_{i}+y_{i+1}}{2}\times \left(t_{i+1}-t_{i}\right)\right]\right.\\ & & \left.+\left[\frac{y_{1}+y_{n}}{2}\times \frac{D}{n\;-1}\right]\right\}\times \frac{n\;-1}{Dn}. \end{array}$$

In this formula, n represents the total number of investigations, D=$\;t_{n}-t_{1}$ represents the total investigation times, and $y_{i}$ and $t_{i}$ represent the disease grade and date of the ith investigation period, respectively.

To achieve a more precise assessment of the BYMD incidence in segregating populations, resistance was scored on February 18, February 24, March 4, and March 15 during the spring of 2020 for ZN1‐F_2_, and on February 21, February 27, March 4, and March 12 in the spring of 2021 for SN2‐F_2_.

### 2.3 BSA‐seq analysis and QTL mapping

Genomic DNA was extracted by the CTAB method (Cheng et al., 2015). A bulked segregant analysis via next‐generation resequencing (BSA‐seq) was applied to identify the genetic signatures of BYMD resistance in ZN1‐F_2_. Two DNA pools were constructed by homogenizing equal quantities of DNA isolated from 30 plants with no visible symptoms (scale 0 for all investigation periods, namely R‐pool) and 30 plants with obvious symptoms (namely S‐pool). Whole‐genome DNA sequencing of two DNA pools and parents (“Zaoshu 3” and “Nongke 1–6”) was performed on the BGISEQ‐500 platform (BGI Tech). Bioinformatics analysis of sequencing begins with the production of raw data. First, we filter the raw data using the SOAPnuke software to remove the adapter sequences and low‐quality reads (Y. Chen et al., 2018). Second, the MEM algorithm of the BWA software was used to align clean data with the barley reference genome Morex_v1 downloaded from EnsemblPlants (Li, 2013; Mascher et al., 2017). Finally, the UnifiedGenotyper function of the GATK software was used for detecting single‐nucleotide polymorphism (SNP) variants, and only SNPs meeting the following standard were retained: (1) parental sites were homozygous and polymorphic, (2) parental sites were not missing, (3) biallelic sites, and (4) GQ values ≥ 50 (McKenna et al., 2010). The Euclidean distance (ED) method and *G* value were used to detect the candidate region between the two pools (H. Liu et al., 2020; Mansfeld & Grumet, 2018). The calculation formula of the ED algorithm was as follows:

(2) $$\mathrm{ED}=\sqrt{{\left(A_{1}-A_{2}\right)}^{2}+{\left(C_{1}-C_{2}\right)}^{2}+{\left(T_{1}-T_{2}\right)}^{2}+{\left(G_{1}-G_{2}\right)}^{2}},$$

where (ATCG)_1_ and (ATCG)_2_ represent the frequency of bases in mixed pools 1 and 2, respectively.

(3) $$G=2\sum_{i=1}^{4}n_{i}\ln\frac{n_{i}}{{\hat{n}}_{i}}.$$

The standard *G*‐statistic serves as a natural metric for data characterization at each SNP, where ${\hat{n}}_{i}$ is the “expected value” for count $n_{i}$. The $n_{i}\;$ represents counts of alleles *A*_0_ and *A*_1_ generated from the sequencing of the segregant bulks. The *G’* value is based on a smoothed version of the standard *G* statistic, considering variations in allele frequency estimates caused by the sampling of segregants to create bulks and the variations introduced during bulk sequencing.

A set of InDel markers evenly distributed within the candidate region were designed with the Primer Premier 5.0 software based on the re‐sequencing data from parents (Table S1). We genotyped F_2_ populations with polymorphic InDel markers and constructed linkage maps of ZN1‐F_2_ and SN2‐F_2_ using the MAP function of the IciMapping 4.2 software (Meng et al., 2015). QTL analysis was performed using the BIP function with the ICIM‐ADD model. Parameters were set as 1 cM per step, PIN = 0.001, and the LOD score (logarithm of the odds) was determined by a 1000‐permutation test.

### 2.4 Genetic background analysis of near‐isogenic lines

The Specific‐Locus Amplified Fragment Sequencing (SLAF‐seq) was performed to design genome‐wide markers on NILs (ZN1‐6011^7D+^ and ZN1‐6012^7D−^) and parents (Sun et al., 2013). In this strategy, the reference genome Morex_v3 was first used for restriction enzyme digestion simulation (Mascher et al., 2021). To obtain evenly distributed markers, *RsaI* was finally selected as the restriction enzyme combination. Digested fragments with length 364–394 bp were defined as SLAF tags. The cut fragments (SLAF tags) were then processed by poly‐A tailing, dual‐index adaptor ligation, polymerase chain reaction (PCR) enrichment, purification, pooling, and size‐selection by electrophoresis. The library after quality checked was processed for sequencing by Illumina platforms. Afterward, the alignment of reads, quality control, and variant calling were consistent with BSA analysis mentioned above.

### 2.5 Transcriptome analysis of near‐isogenic lines

The virus‐infected roots and leaves of ZN1‐6011^7D+^ (abbreviated as IR11 and IL11) and ZN1‐6012^7D−^ (abbreviated as IR12 and IL12) were collected from the disease nursery in March 2024. Meanwhile, the virus‐free roots (FR11, FR12) and leaves (FL11, FL12) of this NIL pair were also collected from disease‐free fields as controls. Three biological replicates were set up in each group. Total RNA was extracted using an RNA isolation kit (TIANGEN) according to the manufacturer's protocol. Purity, concentration, and integrity of RNA sample were examined by NanoDrop, Qubit 2.0, Agilent 2100, and so forth. The cDNA library was constructed by Biomarker Technologies Corporation, and the qualified library was sequenced by the high‐throughput sequencing platform with PE150 mode.

Clean data with high quality were obtained by filtering raw data, which removes adapter sequence and reads with low quality. These clean data were further mapped to predefined reference genome (Morex_v3). fragments per kilobase of transcript per million fragments mapped (FPKM) was applied to measure the expression level of a gene or transcript by StringTie using maximum flow algorithm. Differential expression analysis between groups was performed using the DESeqR software package. Criteria for differentially expressed genes (DEGs) were set as fold change (FC) ≥1.5 and FDR (false discovery rate) < 0.01. Gene Ontology (GO) and Kyoto Encyclopedia of Genes and Genomes (KEGG) pathway enrichment analysis was performed by clusterProfiler. For selected genes, differential expression was validated by quantitative reverse transcription polymerase chain reaction (qRT‐PCR), with *GADPH* serving as a control. Relative transcriptional folds were calculated as 2^−ΔΔCT^.

## 3 RESULTS

### 3.1 Genetic analysis of barley yellow mosaic

**FIGURE 1:**
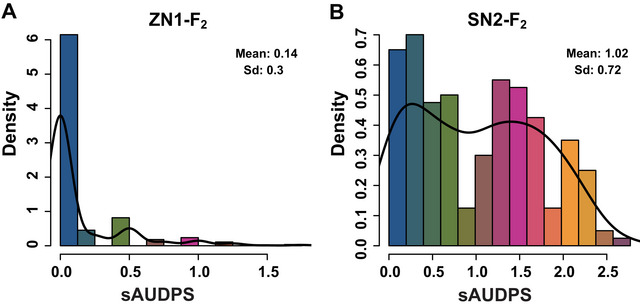
Distribution of standard area under the disease progress stairs (sAUDPs) in the ZN1‐F_2_ (A) and SN2‐F_2_ (B) populations. ZN1‐F_2_ and SN2‐F_2_ represent the F_2_ population derived from the cross “Nongke 1–6” × “Zaoshu 3” and “Nongke 2–6” × “Supi 1,” respectively. Black lines represent the distribution curves.

The plants showing no visible symptoms were considered disease‐resistant, while those with obvious symptoms (disease scale ≥ 1) were considered susceptible (Tables S2 and S3). In the ZN1‐F_2_ population, sAUDPs values indicated a non‐continuous distribution, with most individuals exhibiting a disease‐resistant phenotype and few showing obvious symptoms (Figure 1A). In contrast, the sAUDPs values in the SN2‐F_2_ population exhibited a continuous range, with the mean disease level of individuals (1.02) exceeding Grade 1, and the overall disease incidence is markedly greater than that observed in the ZN1‐F_2_ population (0.14) (Figure 1B). Due to the genetic similarity between “Supi 1” and “Nongke 2–6,” and the continuous distribution of disease levels in their derived F2 segregating population, it suggested that “Nongke 1–6” and “Nongke 2–6” harbor more than one disease resistance QTL from bulbous barley.

### 3.2 Analysis of BSA‐seq and QTL mapping

In this study, a total of 689.13 Gb of clean data were obtained through resequencing, with an average mapping ratio to the reference genome of up to 99%. The average sequencing depth for the parental pools was 28×, and for the disease‐resistant and disease‐susceptible pools, it was 47× (Table S4). After stringent quality control, a total of 8,796,213 high‐quality SNPs were obtained for subsequent BSA mapping analysis.

**FIGURE 2:**
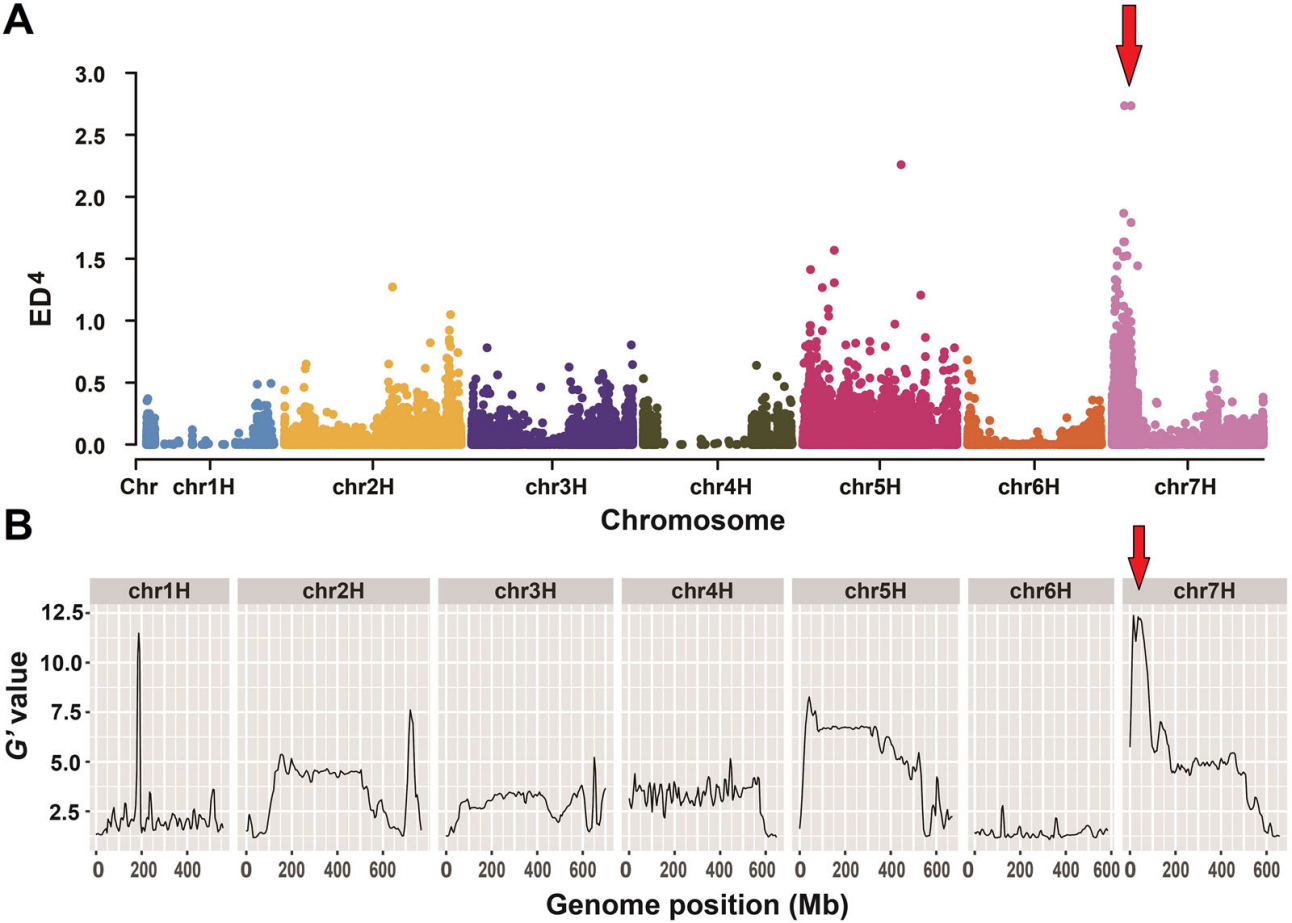
Bulked segregant analysis of the ZN1‐F_2_ population. (A) ED^4^ (Euclidean distance) graph from bulk segregant analysis sequencing (BSA‐seq) analysis between the S‐pool and R‐pool. (B) *G’* value graph from BSA‐seq analysis between the S‐pool and R‐pool. The red arrows represent a co‐located quantitative trait loci (QTL) identified at the top of chromosome 7H by ED method and *G’* value method.

**FIGURE 3:**
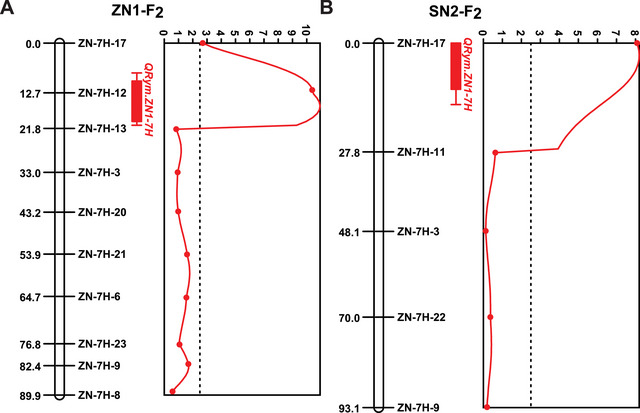
Linkage analysis in the ZN1‐F_2_ (A) and SN2‐F_2_ (B) populations. All the markers were designed according to the parents' resequencing results of ZN1‐F_2_ population, and the genetic maps of these two populations were highly collinear. *QRym.ZN1‐7H* can be identified in both populations.

To mine the resistance QTL for BYMD resistance, the ED method and the *G* value method were used for the detection of significant loci. As a result, within the 0‐ to 100‐Mb interval of chromosome 7H, the two methods jointly identified a significant peak signal, and we called it *QRym.ZN1‐7H* (Figure 2). Based on the resequencing data of “Zaoshu 3” and “Nongke 1–6,” 10 polymorphic Indel markers were developed in this interval, and through genotyping in the ZN1‐F_2_ population, we constructed a genetic map with a length of 89.92 cM. Subsequently, QTL mapping further located *QRym.ZN1‐7H* between markers ZN‐7H‐12 and ZN‐7H‐13, with a genetic interval of 12.67–21.85 cM. This QTL could explain 10.61% of the phenotypic variation with the LOD of 10.93 (Figure 3A and Table 1).

**TABLE 1:** Quantitative trait loci (QTL) mapping for Barley yellow mosaic disease (BYMD) in two different F_2_ populations.

QTL	Chromosome	Left marker	Right marker	Position (cM)	LOD	PVE (%)	Additive effects
*QRym.ZN1‐7H*	7H	ZN‐7H‐12	ZN‐7H‐13	12.67‐21.85	10.93	10.61	0.14
*QRym.ZN1‐7H*	7H	ZN‐7H‐17	ZN‐7H‐11	0.00‐27.77	8.20	19.34	0.37

Abbreviations: LOD, logarithm of the odds; PVE, percent variance explained.

Some polymorphic Indel markers developed between “Zaoshu 3” and “Nongke 1–6” also exhibited polymorphism between “Supi 1” and “Nongke 2–6,” including ZN‐7H‐17, ZN‐7H‐11, ZN‐7H‐3, ZN‐7H‐22, and ZN‐7H‐9. Following genotyping in the SN2‐F_2_ population, we constructed a genetic map with a length of 93.05 cM. Unsurprisingly, a major QTL controlling BYMD was also found between markers ZN‐7H‐17 and ZN‐7H‐11. The genetic interval spanned 0–27.77 cM, explaining 19.34% of the phenotypic variation, with an LOD value of 8.20 (Figure 3B and Table 1). This QTL coincided with the location of *QRym.ZN1‐7H* detected in the ZN1‐F_2_ population, belonging to the same QTL, indicating that *QRym.ZN1‐7H* is highly stable across different genetic backgrounds.

### 3.3 Genetic analysis of NILs

**FIGURE 4:**
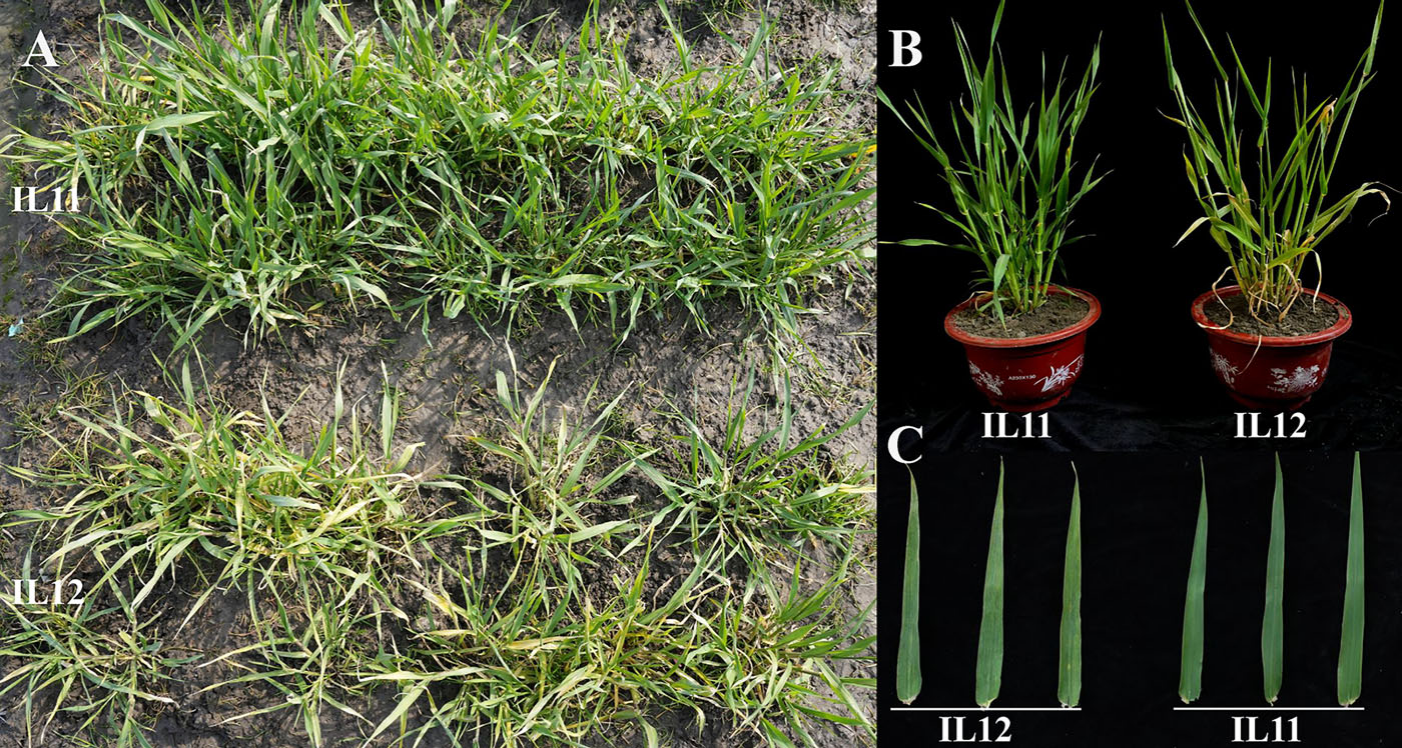
The differences in resistance to barley yellow mosaic disease between near‐isogenic lines. IL11 and IL12 represent the leaves of ZN1‐6011^7D+^ and ZN1‐6012^7D−^ from the disease nursery, respectively.

We screened an F_2_ single plant with a heterozygous genotype in the target interval from the ZN1‐F_2_ population using the QTL flanking markers ZN‐7H‐12 and ZN‐7H‐3. The selected plant was self‐crossed three times in an artificial climate chamber, and then a pair of near‐isogenic lines (ZN1‐6011^7D+^ and ZN1‐6012^7D−^) was obtained. In the field trial, chlorotic spots were significantly more prevalent on the new leaves of ZN1‐6012^7D−^ than those on ZN1‐6011^7D+^ at the seedling stage (Figure 4). Plant height of ZN1‐6012^7D−^ was significantly shorter than that of ZN1‐6011^7D+^ at maturity, but differences in tiller number, grain number, and spike length were not significant (Table 2). SLAF‐seq identified a total of 163,607 SNPs that were polymorphic between the parents. Among these, only 2102 SNPs were polymorphic between the NILs, primarily located within 14–39 Mb on chromosome 7H (Figure S1 and Table S5). This region encompassed the identified BYMD QTL, *QRym.ZN1‐7H*.

**TABLE 2:** Comparison of agronomic traits of near‐isogenic lines.

ID	Plant height (cm)	Spike length (cm)	Grain number per spike	Number of spikes
ZN1‐6011^7D+^	67.93 ± 4.92a	6.97 ± 0.70	15.73 ± 0.96	14.27 ± 6.16
ZN1‐6012^7D‐^	63.67 ± 2.76b	7.01 ± 0.58	15.40 ± 1.35	11.73 ± 3.59

*Note*: Different letters represent significant differences at a level of *p* <0.05.

### 3.4 Differential gene expression of NILs and candidate gene prediction

**FIGURE 5:**
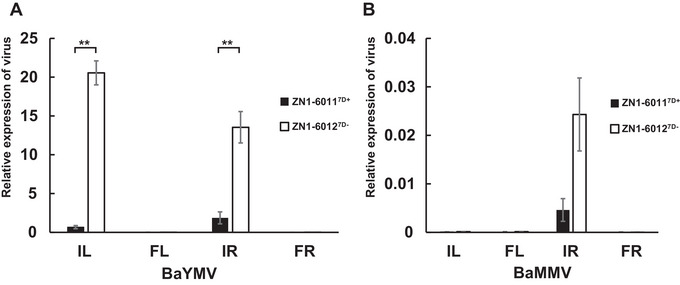
Detection of *Barley yellow mosaic virus* (A) and *Barley mild mosaic virus* (B) in the roots and leaves of near‐isogenic lines. IR and FR represent roots from the disease nursery and disease‐free nursery, respectively. IL and FL represent leaves from the disease nursery and disease‐free nursery, respectively.

Before transcriptome analysis, virus‐based qPCR detection was used to confirm whether samples collected from the disease nursery were infected by BYMD. Primers were designed according to the coat protein sequence of BaYMV and the viral coding sequence of BaMMV, and the expression was normalized to *α‐Tublin* ^[49]^. The results revealed strong infection BaYMV in both roots and leaves of the NILs, while BaMMV was only weakly detected in these tissues (Figure 5), indicating the failure of BaMMV infection. BaYMV levels in the roots and leaves were significantly lower in ZN1‐6011^7D+^ than in ZN1‐6012^7D−^. The presence of disease‐resistant phenotypes and lower virus levels in roots and leaves suggest that this QTL effectively reduces virus infection.

To identify DEGs between ZN1‐6011^7D+^ and ZN1‐6012^7D−^, transcriptome sequencing was performed on the roots and leaves of NILs. A total of 152.70 Gb clean data were obtained, and the principal component analysis and heatmap both showed good repeatability between samples. In the disease nursery, differential expression analysis pinpointed 1751 and 2673 DEGs in the roots and leaves of NILs, respectively (Figure S2). GO enrichment analysis revealed that these genes were significantly enriched in various biological processes. For roots, these processes included carbohydrate metabolism, response to oxidative stress, and hydrogen peroxide catabolic process. In leaves, the processes were translation, protein phosphorylation, and cell surface receptor signaling pathway (Figure S3). KEGG analysis highlighted that root DEGs were significantly enriched in pathways such as carbon metabolism, phenylpropanoid biosynthesis, biosynthesis of amino acids, oxidative phosphorylation, and starch and sucrose metabolism. Meanwhile, leaf DEGs showed substantial enrichment in pathways such as plant–pathogen interaction, ribosome, starch and sucrose metabolism, and plant hormone signal transduction (Figure S4).

### 3.5 Candidate prediction and validation

To predict the candidate genes for *QRym.ZN1‐7H*, we screened DEGs within the differential interval of the near‐isogenic line (14–39 Mb on chromosome 7H). In IR11_IR12, 53 specific DEGs were identified after excluding the common DEGs between FR11_FR12 and IL11_IL12. In IL11_IL12, 35 specific DEGs were identified after excluding the common DEGs between FL11_FL12. Nine DEGs were found to be common under both conditions, which were differentially expressed in both roots and leaves of the NILs (Table 3 and Table S6). In order to verify the accuracy of the results obtained by transcriptome sequencing, eight DEGs were then randomly selected for qRT‐PCR verification (Figure S5). As a result, our transcriptome data proved to be reliable, as RNA‐Seq and qRT‐PCR results were highly consistent.

**TABLE 3:** Nine common differentially expressed genes in the interval of QRym.ZN1‐7H.

Position (bp)	Gene_ID	False discovery rate	log2FC	Annotation
1,456,9126	HORVU.MOREX.r3.7HG0642300	3.68177E‐05	4.05	DUF674 family protein
14,927,866	HORVU.MOREX.r3.7HG0642470	2.39588E‐08	−8.34	ALBINO OR PALE‐GREEN 13
15,558,800	HORVU.MOREX.r3.7HG0642870	5.31714E‐09	8.74	F‐box domain containing protein
17,507,441	HORVU.MOREX.r3.7HG0643990	7.24372E‐08	8.23	P‐loop containing nucleoside triphosphate hydrolases superfamily protein
23,934,819	HORVU.MOREX.r3.7HG0647330	3.45541E‐10	2.04	Mitochondrial metalloendopeptidase OMA1
25,238,994	HORVU.MOREX.r3.7HG0647870	6.29872E‐14	10.19	Esterase/lipase/thioesterase‐like protein
31,427,037	HORVU.MOREX.r3.7HG0650860	8.33498E‐07	−1.35	3′(2′),5′‐bisphosphate nucleotidase
31,645,471	HORVU.MOREX.r3.7HG0650990	3.55775E‐15	2.96	Disease resistance protein (NBS‐LRR class) family
31,866,340	HORVU.MOREX.r3.7HG0651110	2.53115E‐08	−0.92	AT4G29520‐like protein

## 4 DISCUSSION

Host plants and viruses interact in a typical “gene–gene” manner (Li & Shirako, 2015; Li et al., 2016). Although many BYMD resistance QTLs have been identified, the widespread cultivation of accessions carrying major resistance genes has increased selection pressure on BYMD complex. Adaptive improvement and breeding could even accelerate this selection process, enabling new virus strains to easily overcome resistance to existing disease‐resistant sources (Yang, Lüpken, et al., 2014; Yang et al., 2017). Consequently, BYMD must be prevented and controlled by identifying novel resistance sources. A wild self‐incompatible species of barley, bulbous barley is the sole member of the secondary gene pool of cultivated barley and shows resistance to many barley diseases, including BYMD. Innovative breeding practices can be achieved by incorporating novel BYMD resistance genes from bulbous barley. Currently, two resistance loci (*Rym14^Hb^* and *Rym16^Hb^*) from bulbous barley have been reported (Jiang et al., 2020). In this study, the two lines “Nongke 1–6” and “Nongke 2–6” derived from the cross between bulbous barley and “Supi 1” showed immunity to BYMD over multiple years in the disease nursery. In contrast, “Supi 1” was susceptible, indicating that “Nongke 1–6” and “Nongke 2–6” carried the resistance gene from bulbous barley.

Currently, over 22 genes/QTLs associated with BYMD resistance have been identified, predominantly localized on barley chromosomes 3H and 4H. Among these, only two genes, *eIF4E* and *PDIL5‐1*, have been successfully cloned (Stein et al., 2005; Yang, Lüpken, et al., 2014). These QTLs exhibit varying levels of resistance to BaYMV and BaMMV. Some QTLs confer complete immunity or varying degrees of resistance to both viruses, while others are specific to one virus, offering either immunity or differential resistance. To identify resistance loci derived from bulbous barley, two F_2_ populations were constructed using “Nongke 1–6” and “Nongke 2–6” as resistant parents. BSA analysis identified a *G'* signal in the ZN1‐F_2_ population, located in the 0‐ to 100‐Mb region of chromosome 7H. Subsequently, linkage analysis mapped *QRym.ZN1‐7H* between markers ZN‐7H‐12 and ZN‐7H‐13, spanning the 12.67‐ to 21.85‐cM genetic interval. This QTL was also validated in the SN2‐F_2_ population. *QRym.ZN1‐7H*, derived from bulbous barley, is a major QTL for resistance to BYMD, explaining 10.61% (ZN1‐F_2_) and 19.34% (SN2‐F_2_) of the phenotypic variation in different genetic backgrounds, indicating its stability. In contrast to the previously reported two QTLs from bulbous barley, *Rym14^Hb^* and *Rym16^Hb^*, which were located on chromosomes 6H and 2H, respectively (Pidon et al., 2021; Ruge‐Wehling et al., 2006), the QTL identified in this study was located on chromosome 7H. Moreover, the known *rym7t* and *rym2* were in the 213‐ to 369‐Mb interval of chromosome 7H (Takahashi et al., 1973; Takata et al., 2012), which was relatively far from *QRym.ZN1‐7H*, indicating that *QRym.ZN1‐7H* is a novel major QTL for BYMD resistance. It can be reliably used to tag this resistant locus in breeding programs using the highly linked marker. In the disease nursery, the NIL ZN1‐6011^7D+^ carrying *QRym.ZN1‐7H* showed significantly lower levels of BaYMV in both roots and leaves compared to ZN1‐6012^7D−^. Concurrently, chlorotic spots were markedly more prevalent on the newly emerged leaves of ZN1‐6012^7D−^ infected by the virus than those on ZN1‐6011^7D+^ at the seedling stage. It is speculated that *QRym.ZN1‐7H* specifically confers high resistance to the Jiangsu strain of BaYMV.

With the continuous excavation of functional genes, breeding resources gradually change from germplasm to gene resources, and the breeding method also changes from phenotypic selection to gene pyramiding (Nadolska‐Orczyk et al., 2017). High‐throughput RNA‐seq provides a comprehensive transcriptome map to elucidate how certain viruses infect plants. The transcriptomes of virus‐infected and virus‐free plants can be compared for finding genes associated with viral resistance for breeding viral‐resistant crops. This method has been successfully used to identify many candidate genes for resistance to barley Fusarium crown rot (Gao et al., 2023). A BSA‐seq analysis identified 23 DEGs between resistant and susceptible bulks at the wheat yellow mosaic virus resistance locus (*Qupwym.hau‐2DL*), which facilitated the cloning of *TraesCS2D02G513600* (P. Liu et al., 2023). In this study, the genetic background differences of the NILs are predominantly localized within the 7H chromosome interval spanning 14–39 Mb. Within this specific interval, 53 DEGs were identified in the roots, and 35 DEGs were identified in the leaves. Notably, nine genes exhibited differential expressions in both roots and leaves. Among them, *HORVU.MOREX.r3.7HG0650860* and *HORVU.MOREX.r3.7HG0651110* are orthologous to rice *RHL* and *Arabidopsis SES1*, respectively, whose products play a role in plant salt tolerance (Guan et al., 2018; Peng & Verma, 1995). *HORVU.MOREX.r3.7HG0647330* is annotated as mitochondrial metallopeptidase OMA1. Following a stress response in the human body, the activation of protease OMA1 leads to excessive hydrolysis of OPA1, subsequently promoting mitochondrial fragmentation. Persistent activation of this pathway can result in cell death and tissue degeneration, ultimately triggering a series of diseases (Cerveny et al., 2007; Chan, 2006). *HORVU.MOREX.r3.7HG0650990* is annotated as a member of disease resistance protein family (NBS‐LRR class) and is suggested to be the most important candidate for *QRym.ZN1‐7H*. Among the cloned R genes, the NBS‐LRR class constitutes the largest group (Rawandoozi et al., 2022). The N gene, which confers resistance to the tobacco mosaic virus, served as a classic model in the study of plant immunity (Ishibashi & Ishikawa, 2016). The first barley stripe mosaic virus (BSMV) resistance gene *BSR1* has been isolated by map‐based cloning, which encodes a typical CC‐NBS‐LRR protein and recognizes the BSMV‐encoded TGB1 effector (Wu et al., 2022). In wheat, *Ym2* is the major gene for resistance to wheat yellow mosaic disease (WYMV). The candidate gene encodes a CC‐NBS‐LRR protein, and it correlated allelic variation with respect to its sequence with the host's disease response (Mishina et al., 2023).

Previously, using “Zaoshu 3,” “Dan 2,” and “Nongke 1–6” as materials, we explored the genetic responses produced by the plants after BaYMV and/or BaMMV infection at the transcriptional level, and identified many DEGs related to transcription factors, antioxidants, and plant hormones. Here, we explored the regulatory pathways that could be influenced by the BYMD resistance QTL, *QRym.ZN1‐7H*. Upon BYDM infection, DEGs between resistant and susceptible types of the NILs were predominantly enriched in biological processes, including response to oxidative stress, protein phosphorylation, and cell surface receptor signaling pathway. Plants defend against pathogens through innate immunity, activated by pattern–recognition receptors on cell surfaces and intracellular nucleotide‐binding domain leucine‐rich repeat receptors, resulting in pattern‐triggered immunity (Monaghan & Zipfel, 2012) and effector‐triggered immunity (Jones & Dangl, 2006). The two pathways converge on similar downstream outputs, including MAPK cascade, CDPK, burst of reactive oxygen species, and plant hormone signaling (Tsuda & Katagiri, 2010). The biological processes enriched by our differentially expressed genes, including oxidative stress, protein phosphorylation, and cell surface receptor signaling pathways, are highly consistent with these findings, which suggests *QRym.ZN1‐7H* may be involved in signal transduction in plant innate immune response. Our studies will lay a foundation for barley disease resistance genes mining and molecular improvement in breeding practices.

## 5 CONCLUSION

In summary, BSA‐seq identified genomic regions associated with BYMD resistance from bulbous barley. The locus *QRym.ZN1‐7H* emerged as a novel and valuable genetic determinant with a significant impact on BYMD across diverse genetic backgrounds. This locus can be effectively utilized in breeding programs for tagging resistance using highly linked markers. Notably, *QRym.ZN1‐7H* confers high resistance specifically to the Jiangsu strain of BaYMV, leading to a reduction in viral content in the roots and leaves of seedlings and consequently diminishing the occurrence of chlorotic spots on newly emerged leaves. It potentially plays a role in signal transduction within the plant's innate immune response. By comparing the differentially expressed genes between near‐isogenic lines, several candidate genes were identified within this major QTL region. These findings offer critical insights for the fine mapping and isolation of genetic loci associated with BYMD resistance, thereby facilitating the development of barley cultivars with enhanced resistance to BYMD.

## AUTHOR CONTRIBUTIONS

**Yi Hong**: Data curation; writing—review and editing. **Hui Zhou**: Data curation; writing—original draft. **Mengna Zhang**: Investigation. **Yuhang Zhang**: Investigation. **Juan Zhu**: Investigation. **Chao Lv**: Investigation. **Baojian Guo**: Investigation. **Feifei Wang**: Investigation. **Qingliang Li**: Data curation; software; visualization. **Jie Sun**: Data curation; software; visualization. **Rugen Xu**: Funding acquisition; project administration.

## CONFLICT OF INTEREST STATEMENT

The authors declare no conflicts of interest.

## Supporting information




**Figure S1**. Heatmap of density of SNPs at whole genome level among near‐isogenic lines (ZN1‐6011^7D+^ and ZN1‐6012^7D−^). The background identification of near‐isogenic lines based on Morex_v3 reference genome sequence was performed by simplified sequencing (SLAF‐seq). Genomic intervals with continuous differences among near‐isogenic lines were predominantly located between 14–39Mb on chromosome 7H.

**Figure S2**. Volcano diagram of the differentially expressed genes in roots (A) and leaves (B) between near‐isogenic lines from the disease nursery. IR11 represents the roots of ZN1‐6011^7D+^ from the disease nursery. IR12 represents the roots of ZN1‐6012^7D‐^ from the disease nursery. IL11 represents the leaves of ZN1‐6011^7D+^ from the disease nursery. IL12 represents the leaves of ZN1‐6012^7D‐^ from the disease nursery.

**Figure S3**. Biological process of GO enrichment. (A) The top 20 significance terms of biological process of differentially expressed genes enriched in roots of near‐isogenic lines. (B) The top 20 significance terms of biological process of differentially expressed genes enriched in leaves of near‐isogenic lines.

**Figure S4**. KEGG pathway enrichment. (A) The top 20 significance KEGG pathways of differentially expressed genes enriched in roots of near‐isogenic lines. (B) The top 20 significance KEGG pathways of differentially expressed genes enriched in leaves of near‐isogenic lines.

**Figure S5**. Quantitative real‐time PCR validation of 8 randomly selected differentially expressed genes. Dual‐axis graph comparing gene relative expression level obtained from qRT‐PCR (left y‐axis) and RNA‐seq (right y‐axis). The expression trends of RNA‐seq and qRT‐PCR are consistent, it indicates that the results of RNA‐seq are available.

**Table S1**. InDel markers in the QTL mapping interval.

**Table S2**. Phenotype and genotype of ZN1‐F_2_ population.

**Table S3**. Phenotype and genotype of SN2‐F_2_ population.

**Table S4**. Mapping quality statistics.

**Table S5**. Comparison of background differences of near‐isogenic lines.

**Table S6**. Candidate genes in the interval of QRym.ZN1‐7H.

## Data Availability

The datasets supporting the conclusions of this article are included within the article and its additional files.

## References

[tpg220557-bib-0001] Bauer, E. , Weyen, J. , Schiemann, A. , Graner, A. , & Ordon, F. (1997). Molecular mapping of novel resistance genes against Barley Mild Mosaic Virus (BaMMV). Theoretical and Applied Genetics, 95, 1263–1269. 10.1007/s001220050691

[tpg220557-bib-0002] Cerveny, K. L. , Tamura, Y. , Zhang, Z. , Jensen, R. E. , & Sesaki, H. (2007). Regulation of mitochondrial fusion and division. Trends in Cell Biology, 17, 563–569. 10.1016/j.tcb.2007.08.006 17959383

[tpg220557-bib-0003] Chan, D. C. (2006). Mitochondrial fusion and fission in mammals. Annual Review of Cell and Developmental Biology, 22, 79–99. 10.1146/annurev.cellbio.22.010305.104638 16704336

[tpg220557-bib-0004] Chen, J. (1992). Research advance on barley yellow mosaic viruses and their fungal vector *Polymyxa graminis* L. Virologica Sinica, 7, 1.

[tpg220557-bib-0005] Chen, Y. , Chen, Y. , Shi, C. , Huang, Z. , Zhang, Y. , Li, S. , Li, Y. , Ye, J. , Yu, C. , Li, Z. , Zhang, X. , Wang, J. , Yang, H. , Fang, L. , & Chen, Q. (2018). SOAPnuke: A MapReduce acceleration‐supported software for integrated quality control and preprocessing of high‐throughput sequencing data. GigaScience, 7, 1–6. 10.1093/gigascience/gix120 PMC5788068 29220494

[tpg220557-bib-0006] Cheng, X. , Chai, L. , Chen, Z. , Xu, L. , Zhai, H. , Zhao, A. , Peng, H. , Yao, Y. , You, M. , Sun, Q. , & Ni, Z. (2015). Identification and characterization of a high kernel weight mutant induced by gamma radiation in wheat (*Triticum aestivum* L.). BMC Genetics, 16, 127. 10.1186/s12863-015-0285-x 26511975 PMC4625876

[tpg220557-bib-0007] Gao, S. , Jiang, Y. , Zhou, H. , Liu, Y. , Li, H. , Liu, C. , & Zheng, Z. (2023). Fine mapping of a Fusarium crown rot resistant locus on chromosome arm 6HL in barley by exploiting near isogenic lines, transcriptome profiling, and a large near isogenic line‐derived population. Theoretical and Applied Genetics, 136, 1–12. 10.1007/s00122-023-04387-x 37233855 PMC10220118

[tpg220557-bib-0008] Götz, R. , & Friedt, W. (1993). Resistance to the barley yellow mosaic virus complex—Differential genotypic reactions and genetics of BaMMV‐resistance of barley (*Hordeum vulgare* L.). Plant Breeding, 111, 125–131. 10.1111/j.1439-0523.1993.tb00618.x

[tpg220557-bib-0009] Guan, P. , Wang, J. , Li, H. , Xie, C. , Zhang, S. , Wu, C. , Yang, G. , Yan, K. , Huang, J. , & Zheng, C. (2018). SENSITIVE TO SALT1, an endoplasmic reticulum‐localized chaperone, positively regulates salt resistance. Plant Physiology, 178, 1390–1405. 10.1104/pp.18.00840 30287478 PMC6236605

[tpg220557-bib-0010] Humbroich, K. , Jaiser, H. , Schiemann, A. , Devaux, P. , Jacobi, A. , Cselenyi, L. , Habekuss, A. , Friedt, W. , & Ordon, F. (2010). Mapping of resistance against *Barley mild mosaic virus*‐Teik (BaMMV)—An rym5 resistance breaking strain of BaMMV—In the Taiwanese barley (*Hordeum vulgare*) cultivar ‘Taihoku A’. Plant Breeding, 129, 346–348. 10.1111/j.1439-0523.2009.01721.x

[tpg220557-bib-0011] Ishibashi, K. , & Ishikawa, M. (2016). Replication of tobamovirus RNA. Annual Review of Phytopathology, 54, 55–78. 10.1146/annurev-phyto-080615-100217 27296148

[tpg220557-bib-0012] Jiang, C. , Kan, J. , Ordon, F. , Perovic, D. , & Yang, P. (2020). Bymovirus‐induced yellow mosaic diseases in barley and wheat: Viruses, genetic resistances and functional aspects. Theoretical and Applied Genetics, 133, 1623–1640. 10.1007/s00122-020-03555-7 32008056

[tpg220557-bib-0013] Jones, J. D. G. , & Dangl, J. L. (2006). The plant immune system. Nature, 444, 323–329. 10.1038/nature05286 17108957

[tpg220557-bib-0014] Kai, H. , Takata, K. , Tsukazaki, M. , Furusho, M. , & Baba, T. (2012). Molecular mapping of *Rym17*, a dominant and *rym18* a recessive barley yellow mosaic virus (BaYMV) resistance genes derived from *Hordeum vulgare* L. Theoretical and Applied Genetics, 124, 577–583. 10.1007/s00122-011-1730-5 22038435

[tpg220557-bib-0015] Kang, B.‐C. , Yeam, I. , & Jahn, M. M. (2005). Genetics of plant virus resistance. Annual Review of Phytopathology, 43, 581–621. 10.1146/annurev.phyto.43.011205.141140 16078896

[tpg220557-bib-0016] Kanyuka, K. , Druka, A. , Caldwell, D. G. , Tymon, A. , Mccallum, N. , Waugh, R. , & Adams, M. J. (2005). Evidence that the recessive bymovirus resistance locus *rym4* in barley corresponds to the eukaryotic translation initiation factor 4E gene. Molecular Plant Pathology, 6, 449–458. 10.1111/j.1364-3703.2005.00294.x 20565670

[tpg220557-bib-0017] Le Gouis, J. , Devaux, P. , Werner, K. , Hariri, D. , Bahrman, N. , Béghin, D. , & Ordon, F. (2004). *rym15* from the Japanese cultivar Chikurin Ibaraki 1 is a new barley mild mosaic virus (BaMMV) resistance gene mapped on chromosome 6H. Theoretical and Applied Genetics, 108, 1521–1525. 10.1007/s00122-003-1571-y 14747919

[tpg220557-bib-0018] Li, H. (2013). Aligning sequence reads, clone sequences and assembly contigs with BWA‐MEM . arXiv. 10.48550/arXiv.1303.3997

[tpg220557-bib-0019] Li, H. , Kondo, H. , Kühne, T. , & Shirako, Y. (2016). Barley yellow mosaic virus VPg is the determinant protein for breaking eIF4E‐mediated recessive resistance in barley plants. Frontiers in Plant Science, 7, 1–13. 10.3389/fpls.2016.01449 27746794 PMC5043020

[tpg220557-bib-0020] Li, H. , & Shirako, Y. (2015). Association of VPg and eIF4E in the host tropism at the cellular level of *Barley yellow mosaic virus* and *Wheat yellow mosaic virus* in the genus *Bymovirus* . Virology, 476, 159–167. 10.1016/j.virol.2014.12.010 25543966

[tpg220557-bib-0021] Li, H. , Zhang, Y. , Zhong, Y. , Huang, Y. , Xu, R. , Lv, C. , & Huang, Z. (1999). Use of *Hordeum bulbosum* in barley breeding II. Germplasm transfer from *Hordeum bulbosum* to *H. vulgare* . The Crop Journal, 25, 418–425.

[tpg220557-bib-0022] Liu, H. , Zhou, F. , Zhou, T. , Yang, Y. , & Zhao, Y. (2020). Fine mapping of a novel male‐sterile mutant showing wrinkled‐leaf in sesame by BSA‐Seq technology. Industrial Crops and Products, 156, 112862. 10.1016/j.indcrop.2020.112862

[tpg220557-bib-0023] Liu, P. , Shi, C. , Liu, S. , Lei, J. , Lu, Q. , Hu, H. , Ren, Y. , Zhang, N. , Sun, C. , Chen, L. , Jiang, Y. , Feng, L. , Zhang, T. , Zhong, K. , Liu, J. , Zhang, J. , Zhang, Z. , Sun, B. , Chen, J. , … Yang, J. (2023). A papain‐like cysteine protease‐released small signal peptide confers wheat resistance to wheat yellow mosaic virus. Nature Communications, 14, 7773. 10.1038/s41467-023-43643-y PMC10682394 38012219

[tpg220557-bib-0024] Lüpken, T. , Stein, N. , Perovic, D. , Habekuß, A. , Krämer, I. , Hähnel, U. , Steuernagel, B. , Scholz, U. , Zhou, R. , Ariyadasa, R. , Taudien, S. , Platzer, M. , Martis, M. , Mayer, K. , Friedt, W. , & Ordon, F. (2013). Genomics‐based high‐resolution mapping of the BaMMV/BaYMV resistance gene *rym11* in barley (*Hordeum vulgare* L.). Theoretical and Applied Genetics, 126, 1201–1212. 10.1007/s00122-013-2047-3 23456135

[tpg220557-bib-0025] Mansfeld, B. N. , & Grumet, R. (2018). QTLseqr: An R package for bulk segregant analysis with next‐generation sequencing. The The Plant Genome, 11, 1–5. 10.3835/plantgenome2018.01.0006 PMC12810111 30025013

[tpg220557-bib-0026] Mascher, M. , Gundlach, H. , Himmelbach, A. , Beier, S. , Twardziok, S. O. , Wicker, T. , Radchuk, V. , Dockter, C. , Hedley, P. E. , Russell, J. , Bayer, M. , Ramsay, L. , Liu, H. , Haberer, G. , Zhang, X.‐Q. , Zhang, Q. , Barrero, R. A. , Li, L. , Taudien, S. , … Stein, N. (2017). A chromosome conformation capture ordered sequence of the barley genome. Nature, 544, 427–433. 10.1038/nature22043 28447635

[tpg220557-bib-0027] Mascher, M. , Wicker, T. , Jenkins, J. , Plott, C. , Lux, T. , Koh, C. S. , Ens, J. , Gundlach, H. , Boston, L. B. , Tulpová, Z. , Holden, S. , Hernández‐Pinzón, I. , Scholz, U. , Mayer, K. F. X. , Spannagl, M. , Pozniak, C. J. , Sharpe, A. G. , Šimková, H. , Moscou, M. J. , … Stein, N. (2021). Long‐read sequence assembly: A technical evaluation in barley. The Plant Cell, 33, 1888–1906. 10.1093/plcell/koab077 33710295 PMC8290290

[tpg220557-bib-0028] McKenna, A. , Hanna, M. , Banks, E. , Sivachenko, A. , Cibulskis, K. , Kernytsky, A. , Garimella, K. , Altshuler, D. , Gabriel, S. , Daly, M. , & DePristo, M. A. (2010). The Genome Analysis Toolkit: A MapReduce framework for analyzing next‐generation DNA sequencing data. Genome Research, 20, 1297–1303. 10.1101/gr.107524.110 20644199 PMC2928508

[tpg220557-bib-0029] Meng, L. , Li, H. , Zhang, L. , & Wang, J. (2015). QTL IciMapping: Integrated software for genetic linkage map construction and quantitative trait locus mapping in biparental populations. Crop Journal, 3, 269–283. 10.1016/j.cj.2015.01.001

[tpg220557-bib-0030] Mishina, K. , Suzuki, T. , Oono, Y. , Yamashita, Y. , Zhu, H. , Ogawa, T. , Ohta, M. , Doman, K. , Xu, W. , Takahashi, D. , Miyazaki, T. , Tagiri, A. , Soma, C. , Horita, H. , Nasuda, S. , De Oliveira, R. , Paux, E. , Chen, G. , Pourkheirandish, M. , … Komatsuda, T. (2023). Wheat *Ym2* originated from *Aegilops sharonensis* and confers resistance to soil‐borne *Wheat yellow mosaic virus* infection to the roots. Proceedings of the National Academy of Sciences, 120, 2017. 10.1073/pnas.2214968120 PMC10089197 36897977

[tpg220557-bib-0031] Monaghan, J. , & Zipfel, C. (2012). Plant pattern recognition receptor complexes at the plasma membrane. Current Opinion in Plant Biology, 15, 349–357. 10.1016/j.pbi.2012.05.006 22705024

[tpg220557-bib-0032] Nadolska‐Orczyk, A. , Rajchel, I. K. , Orczyk, W. , & Gasparis, S. (2017). Major genes determining yield‐related traits in wheat and barley. Theoretical and Applied Genetics, 130, 1081–1098. 10.1007/s00122-017-2880-x 28314933 PMC5440550

[tpg220557-bib-0033] Okada, Y. , Kashiwazaki, S. , Kanatani, R. , Arai, S. , & Ito, K. (2003). Effects of barley yellow mosaic disease resistant gene *rym1* on the infection by strains of *Barley yellow mosaic virus* and *Barley mild mosaic virus* . Theoretical and Applied Genetics, 106, 181–189. 10.1007/s00122-002-1019-9 12582842

[tpg220557-bib-0034] Pan, Y. , Hong, Y. , Xu, X. , Luan, H. , Zhu, J. , Lv, C. , Guo, B. , Shen, H. , & Xu, R. (2022). Haplotypes of HvPDIL5‐1 and HvEIF4E and resistance to yellow mosaic disease of different barley varieties (Lines). Journal of Triticeae Crops, 42, 153–162.

[tpg220557-bib-0035] Pan, Y. , Zhu, J. , Hong, Y. , Zhang, M. , Lv, C. , Guo, B. , Shen, H. , Xu, X. , & Xu, R. (2021). Identification of novel QTL contributing to barley yellow mosaic resistance in wild barley (*Hordeum vulgare* spp. *spontaneum*). BMC Plant Biology, 21, 1–11. 10.1186/s12870-021-03321-x 34823470 PMC8613928

[tpg220557-bib-0036] Peng, Z. , & Verma, D. P. S. (1995). A rice *HAL_2_ *‐like gene encodes a Ca^2+^‐sensitive 3′(2′),5′‐diphosphonucleoside 3′(2′)‐phosphohydrolase and complements yeast met22 and *Escherichia coli* cysQ mutations. Journal of Biological Chemistry, 270, 29105–29110. 10.1074/jbc.270.49.29105 7493934

[tpg220557-bib-0037] Perovic, D. , Krämer, I. , Habekuss, A. , Perner, K. , Pickering, R. , Proeseler, G. , Kanyuka, K. , & Ordon, F. (2014). Genetic analyses of BaMMV/BaYMV resistance in barley accession HOR4224 result in the identification of an allele of the translation initiation factor 4e (*Hv‐eIF4E*) exclusively effective against *Barley mild mosaic virus* (BaMMV). Theoretical and Applied Genetics, 127, 1061–1071. 10.1007/s00122-014-2279-x 24522725

[tpg220557-bib-0038] Pidon, H. , Wendler, N. , Habekuβ, A. , Maasberg, A. , Ruge‐Wehling, B. , Perovic, D. , Ordon, F. , & Stein, N. (2021). High‐resolution mapping of *Rym14^Hb^ *, a wild relative resistance gene to barley yellow mosaic disease. Theoretical and Applied Genetics, 134, 823–833. 10.1007/s00122-020-03733-7 33263784 PMC7925471

[tpg220557-bib-0039] Plumb, R. T. , Lennon, E. A. , & Gutteridge, R. A. (1986). The effects of infection by barley yellow mosaic virus on the yield and components of yield of barley. Plant Pathology, 35, 314–318. 10.1111/j.1365-3059.1986.tb02020.x

[tpg220557-bib-0040] Rawandoozi, Z. J. , Young, E. L. , Yan, M. , Noyan, S. , Fu, Q. , Hochhaus, T. , Rawandoozi, M. Y. , Klein, P. E. , Byrne, D. H. , & Riera‐Lizarazu, O. (2022). QTL mapping and characterization of black spot disease resistance using two multi‐parental diploid rose populations. Horticulture Research, 9, uhac183. 10.1093/hr/uhac183 37064269 PMC10101596

[tpg220557-bib-0041] Ruge, B. , Linz, A. , Pickering, R. , Proeseler, G. , Greif, P. , & Wehling, P. (2003). Mapping of *Rym14^Hb^ *, a gene introgressed from *Hordeum bulbosum* and conferring resistance to BaMMV and BaYMV in barley. Theoretical and Applied Genetics, 107, 965–971. 10.1007/s00122-003-1339-4 12830389

[tpg220557-bib-0042] Ruge‐Wehling, B. , Linz, A. , Habekuß, A. , & Wehling, P. (2006). Mapping of *Rym16^Hb^ *, the second soil‐borne virus‐resistance gene introgressed from *Hordeum bulbosum* . Theoretical and Applied Genetics, 113, 867–873. 10.1007/s00122-006-0345-8 16838136

[tpg220557-bib-0043] Saeki, K. , Miyazaki, C. , Hirota, N. , Saito, A. , Ito, K. , & Konishi, T. (1999). RFLP mapping of BaYMV resistance gene *rym3* in barley (*Hordeum vulgare*). Theoretical and Applied Genetics, 99, 727–732. 10.1007/s001220051290 22665211

[tpg220557-bib-0044] Shi, L. , Jiang, C. , He, Q. , Habekuß, A. , Ordon, F. , Luan, H. , Shen, H. , Liu, J. , Feng, Z. , Zhang, J. , & Yang, P. (2019). Bulked segregant RNA‐sequencing (BSR‐seq) identified a novel rare allele of eIF4E effective against multiple isolates of BaYMV/BaMMV. Theoretical and Applied Genetics, 132, 1777–1788. 10.1007/s00122-019-03314-3 30815718

[tpg220557-bib-0045] Simko, I. , Ochoa, O. E. , Pel, M. A. , Tsuchida, C. , Font i Forcada, C. , Hayes, R. J. , Truco, M.‐J. , Antonise, R. , Galeano, C. H. , & Michelmore, R. W. (2015). Resistance to downy mildew in lettuce ‘La Brillante’ is conferred by *Dm50* gene and multiple QTL. Phytopathology, 105, 1220–1228. 10.1094/PHYTO-02-15-0057-R 25915441

[tpg220557-bib-0046] Simko, I. , & Piepho, H. P. (2012). The area under the disease progress stairs: Calculation, advantage, and application. Phytopathology, 102, 381–389. 10.1094/PHYTO-07-11-0216 22122266

[tpg220557-bib-0047] Stein, N. , Perovic, D. , Kumlehn, J. , Pellio, B. , Stracke, S. , Streng, S. , Ordon, F. , & Graner, A. (2005). The eukaryotic translation initiation factor 4E confers multiallelic recessive *Bymovirus* resistance in *Hordeum vulgare* (L.). The Plant Journal, 42, 912–922. 10.1111/j.1365-313X.2005.02424.x 15941403

[tpg220557-bib-0048] Sun, X. , Liu, D. , Zhang, X. , Li, W. , Liu, H. , Hong, W. , Jiang, C. , Guan, N. , Ma, C. , Zeng, H. , Xu, C. , Song, J. , Huang, L. , Wang, C. , Shi, J. , Wang, R. , Zheng, X. , Lu, C. , Wang, X. , & Zheng, H. (2013). SLAF‐seq: An efficient method of large‐scale de novo SNP discovery and genotyping using high‐throughput sequencing. PLoS ONE, 8, e58700. 10.1371/journal.pone.0058700 23527008 PMC3602454

[tpg220557-bib-0049] Takahashi, R. , Hayashi, J. , Inouye, T. , Moriya, I. , & Hirao, C. (1973). Studies on resistance to yellow mosaic disease in barely . *I.* Tests for varietal reactions and genetic analysis of resistance to the disease*. CiNii. https://cir.nii.ac.jp/crid/1050002213154023552?lang=en

[tpg220557-bib-0050] Takata, K. , Kai, H. , Uchimura, Y. , Tsukazaki, M. , Furusho, M. , & Baba, T. (2012). Selection of DNA markers closely linked to the resistance gene *rym7t* against Barley yellow mosaic disease. Breeding Research, 14, 43–49. 10.1270/jsbbr.14.43

[tpg220557-bib-0051] Tsuda, K. , & Katagiri, F. (2010). Comparing signaling mechanisms engaged in pattern‐triggered and effector‐triggered immunity. Current Opinion in Plant Biology, 13, 459–465. 10.1016/j.pbi.2010.04.006 20471306

[tpg220557-bib-0052] Werner, K. , Friedt, W. , Laubach, E. , Waugh, R. , & Ordon, F. (2003). Dissection of resistance to soil‐borne yellow‐mosaic‐inducing viruses of barley (BaMMV, BaYMV, BaYMV‐2) in a complex breeders' cross by means of SSRs and simultaneous mapping of BaYMV/BaYMV‐2 resistance of var. ‘Chikurin Ibaraki 1’. Theoretical and Applied Genetics, 106, 1425–1432. 10.1007/s00122-002-1188-6 12750785

[tpg220557-bib-0053] Werner, K. , Friedt, W. , & Ordon, F. (2005). Strategies for pyramiding resistance genes against the Barley Yellow Mosaic Virus complex (BaMMV, BaYMV, BaYMV‐2). Molecular Breeding, 16, 45–55. 10.1007/s11032-005-3445-2

[tpg220557-bib-0054] Wu, Q. , Cui, Y. , Jin, X. , Wang, G. , Yan, L. , Zhong, C. , Yu, M. , Li, W. , Wang, Y. , Wang, L. , Wang, H. , Dang, C. , Zhang, X. , Chen, Y. , Zhang, P. , Zhao, X. , Wu, J. , Fu, D. , Xia, L. , … Liu, Z. (2022). The CC‐NB‐LRR protein BSR1 from *Brachypodium* confers resistance to *Barley stripe mosaic virus* in gramineous plants by recognizing TGB1 movement protein. New Phytologist, 236, 2233–2248. 10.1111/nph.18457 36059081

[tpg220557-bib-0055] Yang, P. , Habekuß, A. , Hofinger, B. J. , Kanyuka, K. , Kilian, B. , Graner, A. , Ordon, F. , & Stein, N. (2017). Sequence diversification in recessive alleles of two host factor genes suggests adaptive selection for bymovirus resistance in cultivated barley from East Asia. Theoretical and Applied Genetics, 130, 331–344. 10.1007/s00122-016-2814-z 27830284 PMC5263206

[tpg220557-bib-0056] Yang, P. , Habekuß, A. , Ordon, F. , & Stein, N. (2014). Analysis of bymovirus resistance genes on proximal barley chromosome 4HL provides the basis for precision breeding for BaMMV/BaYMV resistance. Theoretical and Applied Genetics, 127, 1625–1634. 10.1007/s00122-014-2324-9 24849455

[tpg220557-bib-0057] Yang, P. , Lüpken, T. , Habekuss, A. , Hensel, G. , Steuernagel, B. , Kilian, B. , Ariyadasa, R. , Himmelbach, A. , Kumlehn, J. , Scholz, U. , Ordon, F. , & Stein, N. (2014). Protein disulfide isomerase like 5‐1 is a susceptibility factor to plant viruses. Proceedings of the National Academy of Sciences of the United States of America, 111, 2104–2109. 10.1073/pnas.1320362111 24481254 PMC3926060

[tpg220557-bib-0058] Yang, P. , Perovic, D. , Habekuß, A. , Zhou, R. , Graner, A. , Ordon, F. , & Stein, N. (2013). Gene‐based high‐density mapping of the gene rym7 conferring resistance to *Barley mild mosaic virus* (BaMMV). Molecular Breeding, 32, 27–37. 10.1007/s11032-013-9842-z

[tpg220557-bib-0059] Zhang, Y. , Shen, C. , Li, G. , Shi, J. , Yuan, Y. , Ye, L. , Song, Q. , Shi, J. , & Zhang, D. (2024). MADS1‐regulated lemma and awn development benefits barley yield. Nature Communications, 15, 301. 10.1038/s41467-023-44457-8 PMC10770128 38182608

