# Supplementary material for: Identification and characterization of a novel QTL for barley yellow mosaic disease resistance from bulbous barley
Source: Plant Genome. 2025 Jan 13;18(1):e20557. doi: 10.1002/tpg2.20557 (PMC11726411; doi:10.1002/tpg2.20557)
Supplement: Supplementary file 1 [file TPG2-18-e20557-s002.pdf]

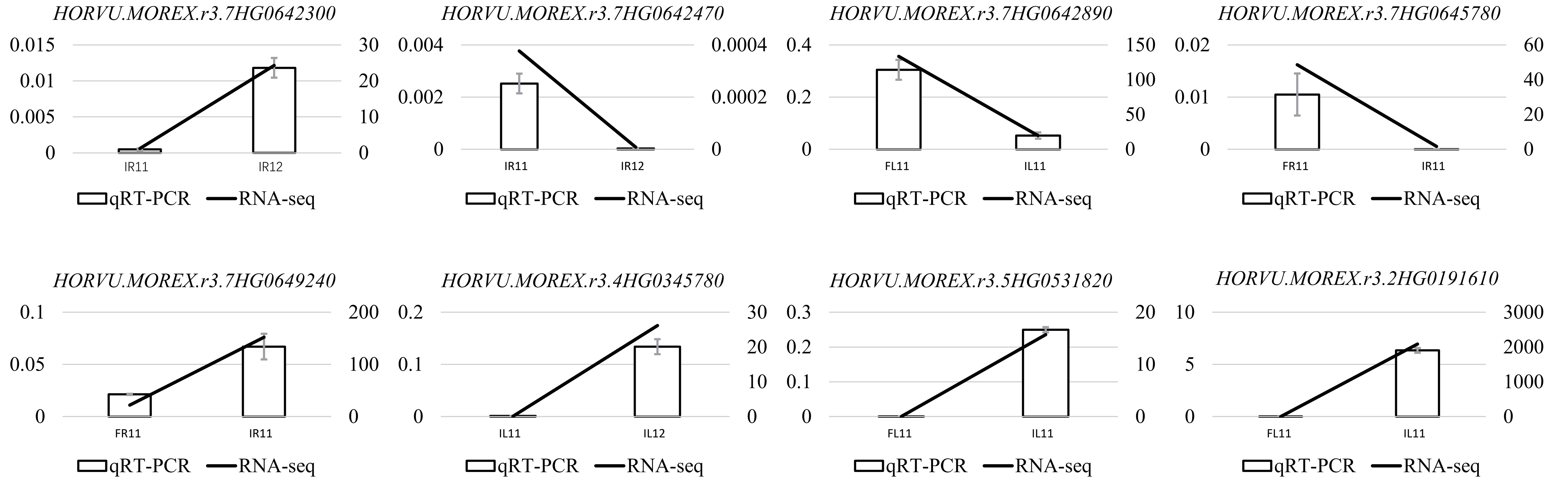
HORVU.MOREX.r3.7HG0642300
0.015
0.01
0.005
0
30
20
10
0
IR11
IR12
qRT-PCR
RNA-seq
HORVU.MOREX.r3.7HG0642470
0.004
0.002
0
0.0004
0.0002
0
IR11
IR12
qRT-PCR
RNA-seq
HORVU.MOREX.r3.7HG0642890
0.4
0.2
0
150
100
50
0
FL11
IL11
qRT-PCR
RNA-seq
HORVU.MOREX.r3.7HG0645780
0.02
0.01
0
60
40
20
0
FR11
IR11
qRT-PCR
RNA-seq
HORVU.MOREX.r3.7HG0649240
0.1
0.05
0
200
100
0
FR11
IR11
qRT-PCR
RNA-seq
HORVU.MOREX.r3.4HG0345780
0.2
0.1
0
30
20
10
0
IL11
IL12
qRT-PCR
RNA-seq
HORVU.MOREX.r3.5HG0531820
0.3
0.2
0.1
0
20
10
0
FL11
IL11
qRT-PCR
RNA-seq
HORVU.MOREX.r3.2HG0191610
10
5
0
3000
2000
1000
0
FL11
IL11
qRT-PCR
RNA-seq
