# Supplementary material for: Identification and characterization of a novel QTL for barley yellow mosaic disease resistance from bulbous barley
Source: Plant Genome. 2025 Jan 13;18(1):e20557. doi: 10.1002/tpg2.20557 (PMC11726411; doi:10.1002/tpg2.20557)
Supplement: Supplementary file 2 [file TPG2-18-e20557-s007.pdf]

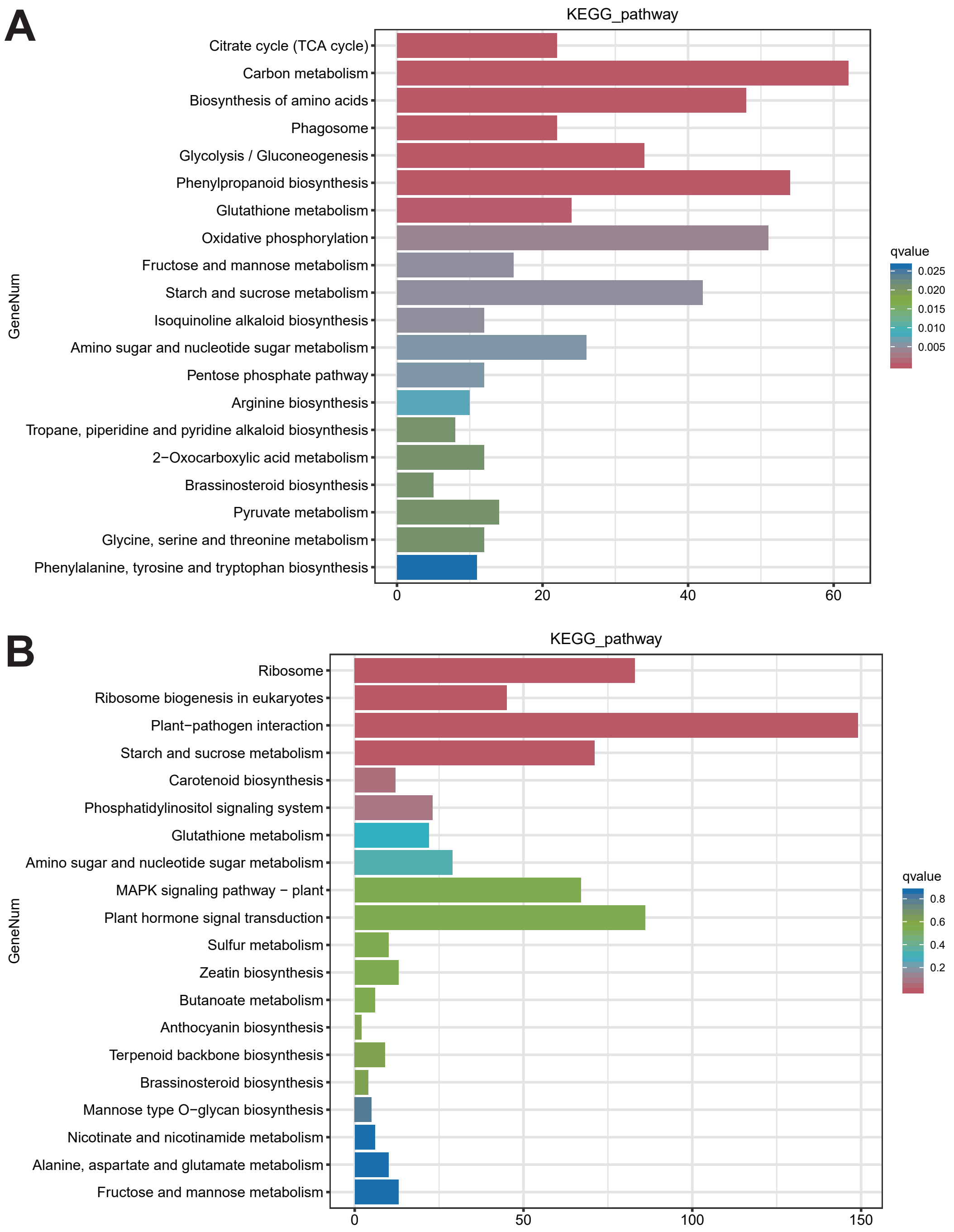
A
KEGG_pathway
Citrate cycle (TCA cycle)
Carbon metabolism
Biosynthesis of amino acids
Phagosome
Glycolysis / Gluconeogenesis
Phenylpropanoid biosynthesis
Glutathione metabolism
Oxidative phosphorylation
Fructose and mannose metabolism
Starch and sucrose metabolism
Isoquinoline alkaloid biosynthesis
Amino sugar and nucleotide sugar metabolism
Pentose phosphate pathway
Arginine biosynthesis
Tropane, piperidine and pyridine alkaloid biosynthesis
2−Oxocarboxylic acid metabolism
Brassinosteroid biosynthesis
Pyruvate metabolism
Glycine, serine and threonine metabolism
Phenylalanine, tyrosine and tryptophan biosynthesis
GeneNum
0
20
40
60
qvalue
0.025
0.020
0.015
0.010
0.005
B
KEGG_pathway
Ribosome
Ribosome biogenesis in eukaryotes
Plant−pathogen interaction
Starch and sucrose metabolism
Carotenoid biosynthesis
Phosphatidylinositol signaling system
Glutathione metabolism
Amino sugar and nucleotide sugar metabolism
MAPK signaling pathway − plant
Plant hormone signal transduction
Sulfur metabolism
Zeatin biosynthesis
Butanoate metabolism
Anthocyanin biosynthesis
Terpenoid backbone biosynthesis
Brassinosteroid biosynthesis
Mannose type O−glycan biosynthesis
Nicotinate and nicotinamide metabolism
Alanine, aspartate and glutamate metabolism
Fructose and mannose metabolism
GeneNum
0
50
100
150
qvalue
0.8
0.6
0.4
0.2
