# Supplementary material for: Identification and characterization of a novel QTL for barley yellow mosaic disease resistance from bulbous barley
Source: Plant Genome. 2025 Jan 13;18(1):e20557. doi: 10.1002/tpg2.20557 (PMC11726411; doi:10.1002/tpg2.20557)
Supplement: Supplementary file 3 [file TPG2-18-e20557-s008.pdf]

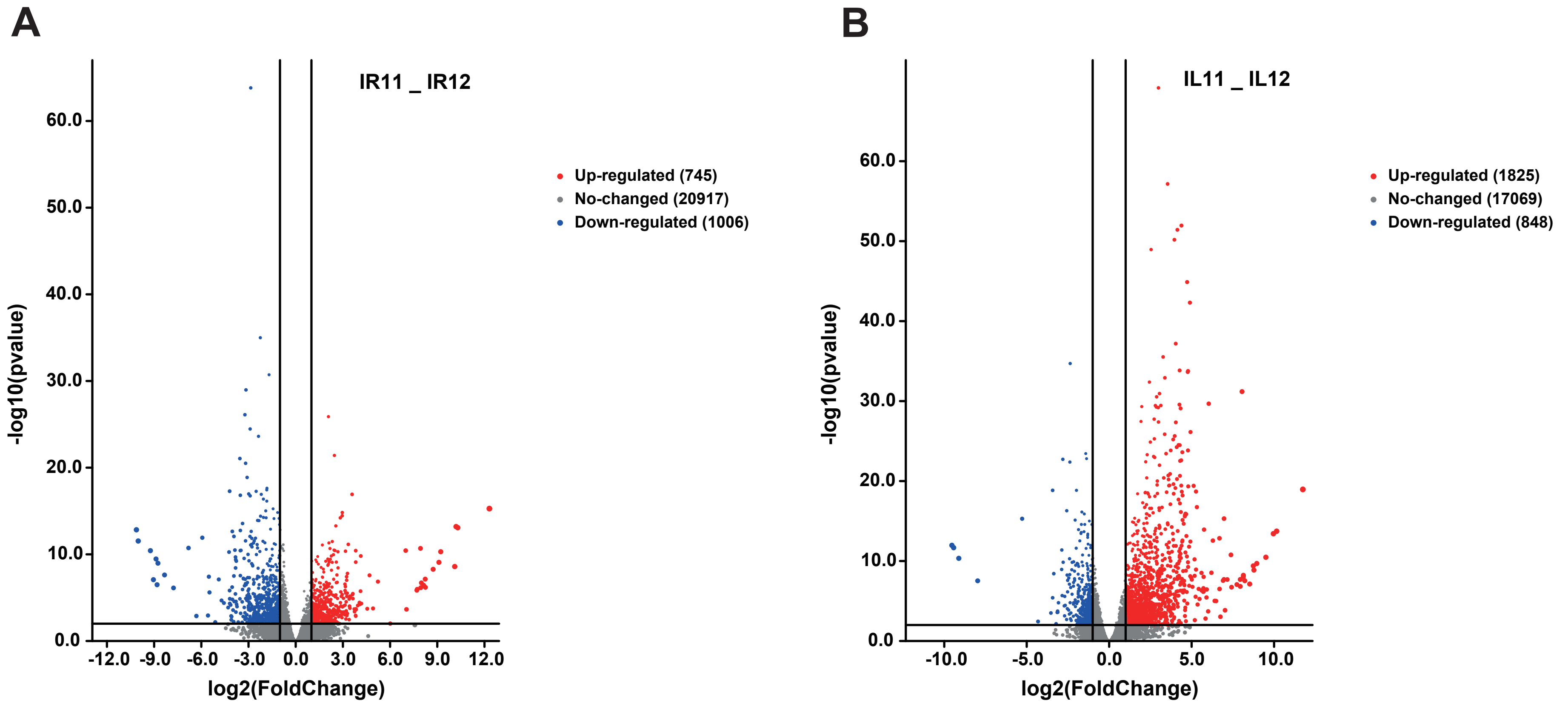
A
IR11 _ IR12
Up-regulated (745)
No-changed (20917)
Down-regulated (1006)
-log10(pvalue)
0.0
10.0
20.0
30.0
40.0
50.0
60.0
-12.0
-9.0
-6.0
-3.0
0.0
3.0
6.0
9.0
12.0
log2(FoldChange)
B
IL11 _ IL12
Up-regulated (1825)
No-changed (17069)
Down-regulated (848)
-log10(pvalue)
0.0
10.0
20.0
30.0
40.0
50.0
60.0
-10.0
-5.0
0.0
5.0
10.0
log2(FoldChange)
