# Supplementary material for: Identification and characterization of a novel QTL for barley yellow mosaic disease resistance from bulbous barley
Source: Plant Genome. 2025 Jan 13;18(1):e20557. doi: 10.1002/tpg2.20557 (PMC11726411; doi:10.1002/tpg2.20557)
Supplement: Supplementary file 4 [file TPG2-18-e20557-s009.pdf]

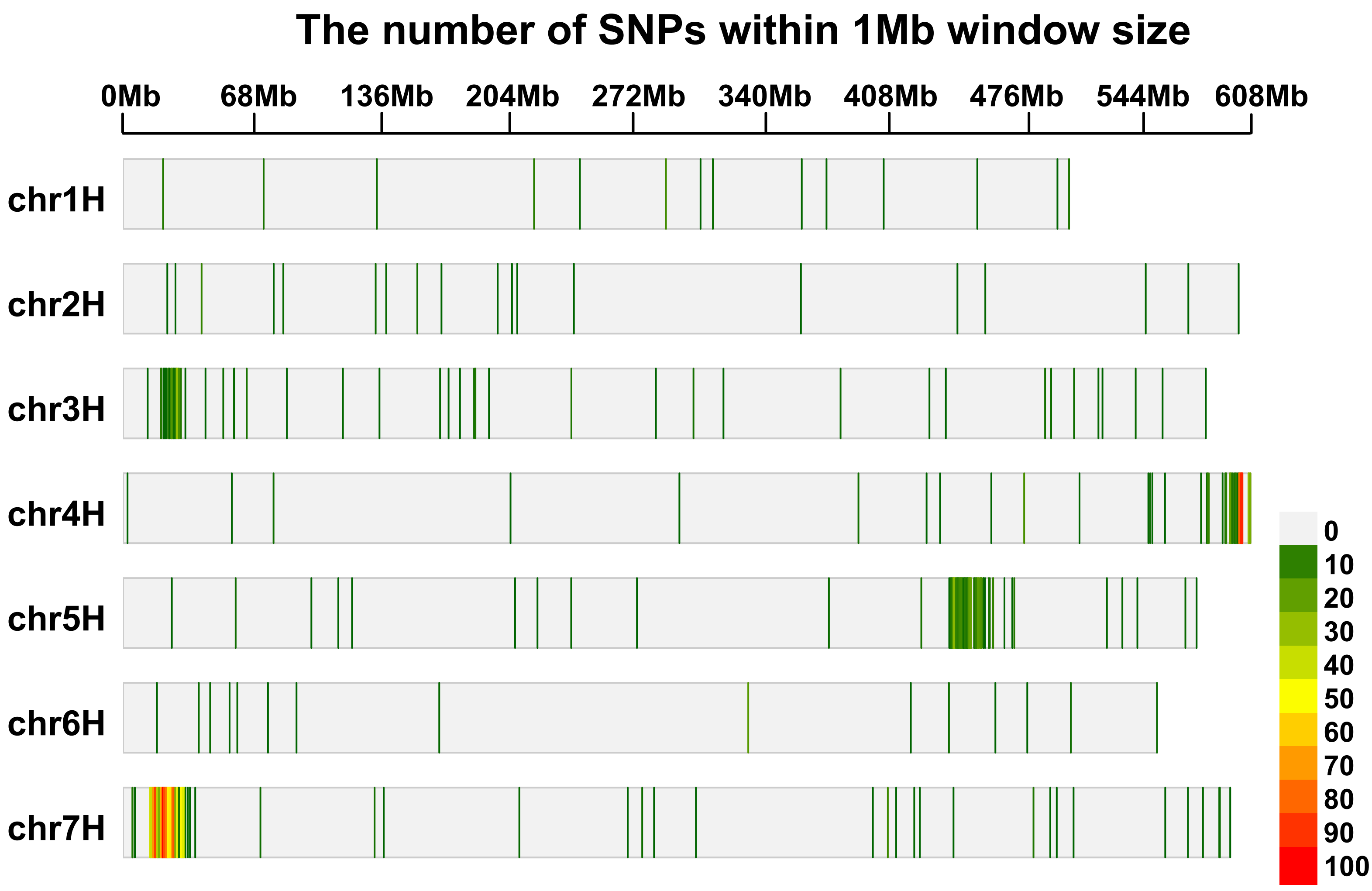
The number of SNPs within 1Mb window size
0Mb
68Mb
136Mb
204Mb
272Mb
340Mb
408Mb
476Mb
544Mb
608Mb
chr1H
chr2H
chr3H
chr4H
chr5H
chr6H
chr7H
0
10
20
30
40
50
60
70
80
90
100
