# Supplementary material for: Identification and characterization of a novel QTL for barley yellow mosaic disease resistance from bulbous barley
Source: Plant Genome. 2025 Jan 13;18(1):e20557. doi: 10.1002/tpg2.20557 (PMC11726411; doi:10.1002/tpg2.20557)
Supplement: Supplementary file 5 [file TPG2-18-e20557-s010.pdf]

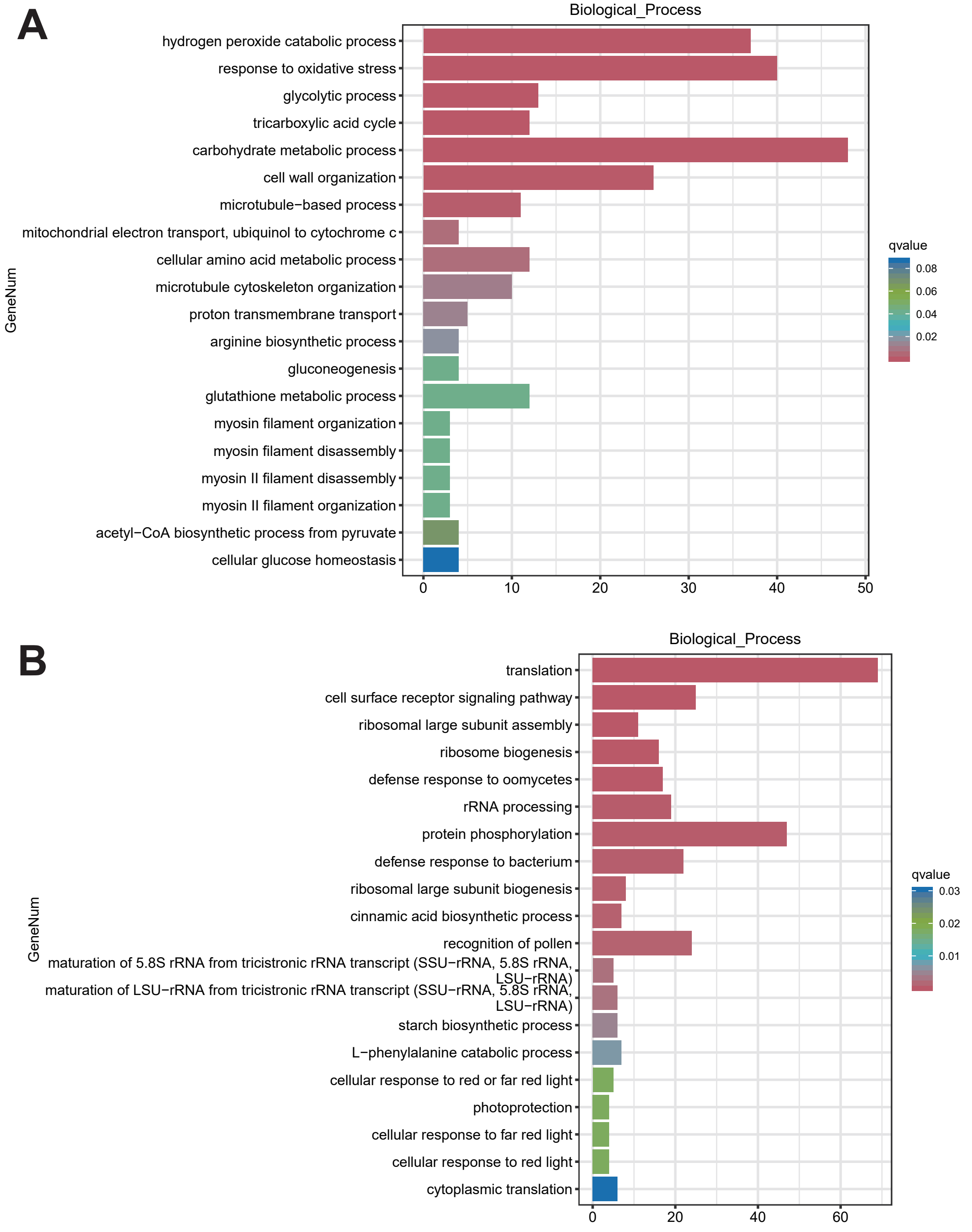
A
Biological_Process
hydrogen peroxide catabolic process
response to oxidative stress
glycolytic process
tricarboxylic acid cycle
carbohydrate metabolic process
cell wall organization
microtubule−based process
mitochondrial electron transport, ubiquinol to cytochrome c
cellular amino acid metabolic process
microtubule cytoskeleton organization
proton transmembrane transport
arginine biosynthetic process
gluconeogenesis
glutathione metabolic process
myosin filament organization
myosin filament disassembly
myosin II filament disassembly
myosin II filament organization
acetyl−CoA biosynthetic process from pyruvate
cellular glucose homeostasis
GeneNum
0
10
20
30
40
50
qvalue
0.08
0.06
0.04
0.02
B
Biological_Process
translation
cell surface receptor signaling pathway
ribosomal large subunit assembly
ribosome biogenesis
defense response to oomycetes
rRNA processing
protein phosphorylation
defense response to bacterium
ribosomal large subunit biogenesis
cinnamic acid biosynthetic process
recognition of pollen
maturation of 5.8S rRNA from tricistronic rRNA transcript (SSU−rRNA, 5.8S rRNA, LSU−rRNA)
maturation of LSU−rRNA from tricistronic rRNA transcript (SSU−rRNA, 5.8S rRNA, LSU−rRNA)
starch biosynthetic process
L−phenylalanine catabolic process
cellular response to red or far red light
photoprotection
cellular response to far red light
cellular response to red light
cytoplasmic translation
GeneNum
0
20
40
60
qvalue
0.03
0.02
0.01
