# Supplementary figures and images for: Identification and characterization of a novel QTL for barley yellow mosaic disease resistance from bulbous barley
Source: Plant Genome. 2025 Jan 13;18(1):e20557. doi: 10.1002/tpg2.20557 (PMC11726411; doi:10.1002/tpg2.20557)

# The number of SNPs within 1Mb window size

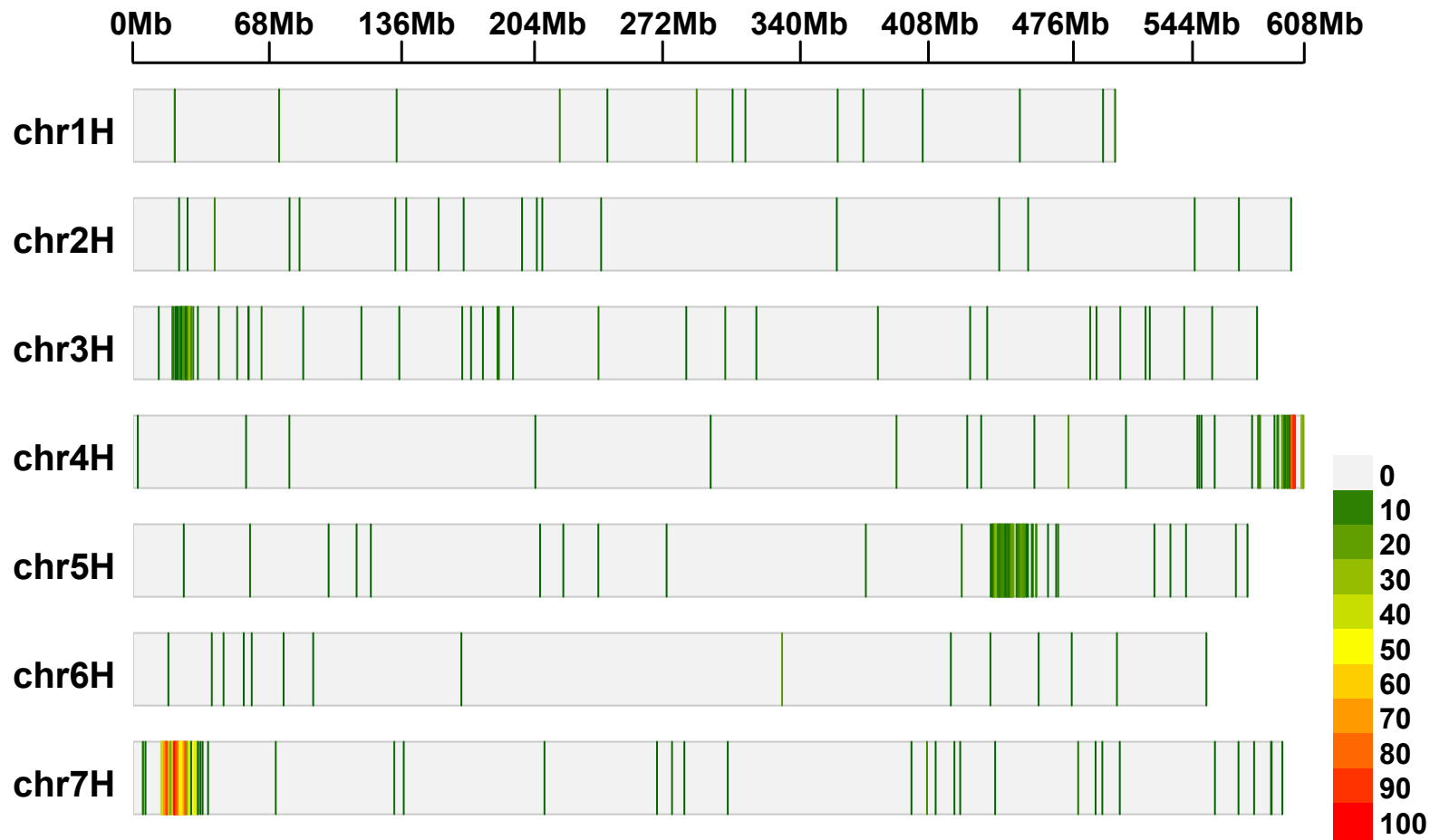

Supplement: Supplementary file 1 — Figure S1. Heatmap of density of SNPs at whole genome level among near‐isogenic lines (ZN1‐60117D+ and ZN1‐60127D−). The background identification of near‐isogenic lines based on Morex_v3 reference genome sequence was performed by simplified sequencing (SLAF‐seq). Genomic intervals with continuous differences among near‐isogenic lines were predominantly located between 14–39Mb on chromosome 7H. [file TPG2-18-e20557-s009.pdf]

**A**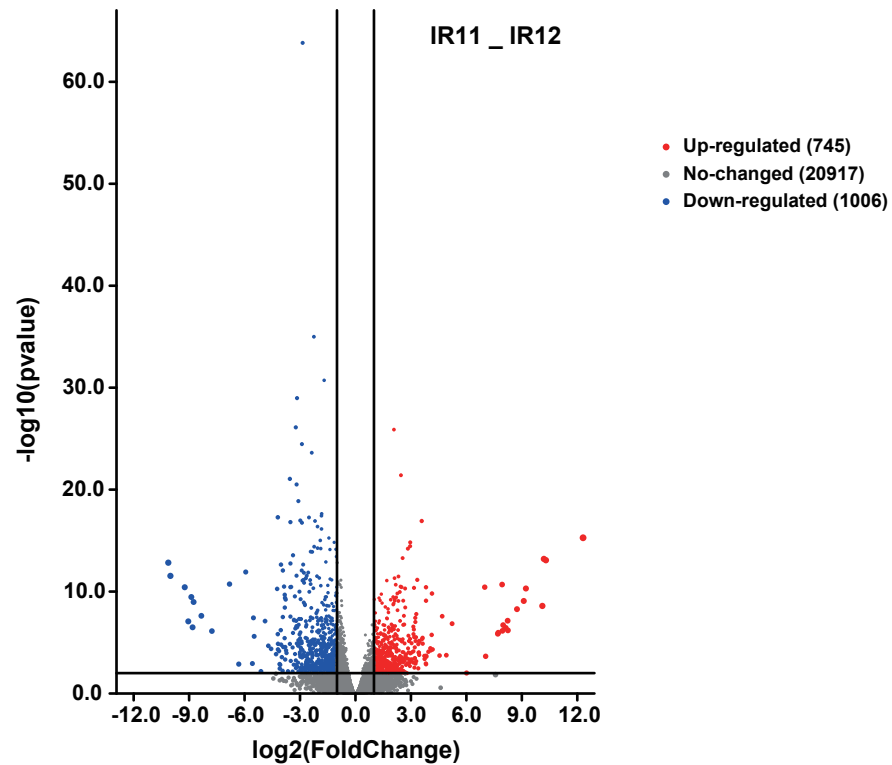**B**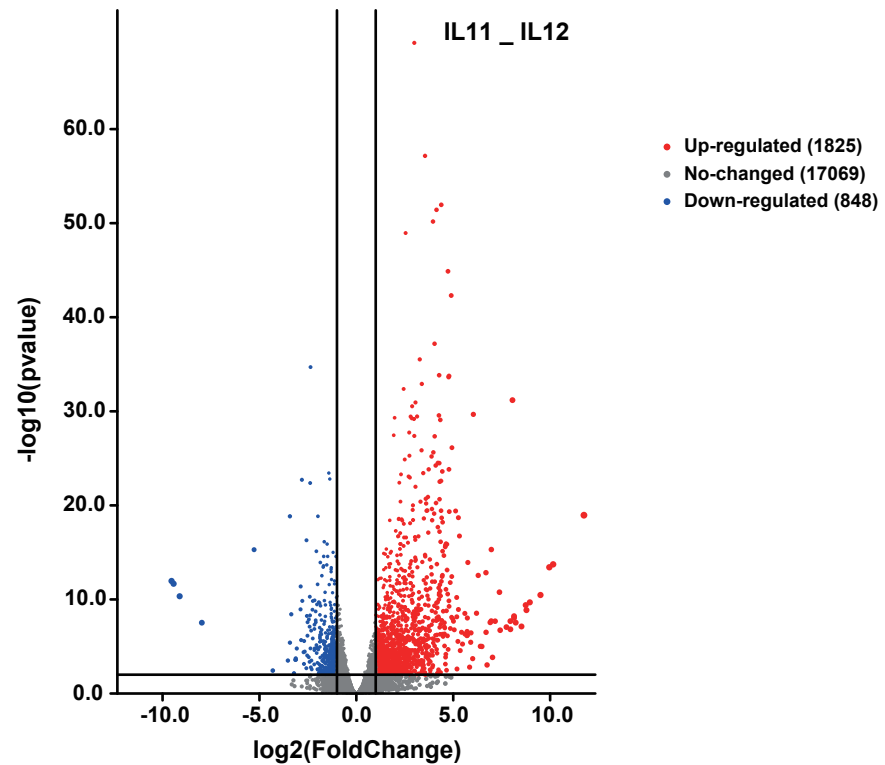

Supplement: Supplementary file 2 — Figure S2. Volcano diagram of the differentially expressed genes in roots (A) and leaves (B) between near‐isogenic lines from the disease nursery. IR11 represents the roots of ZN1‐60117D+ from the disease nursery. IR12 represents the roots of ZN1‐60127D‐ from the disease nursery. IL11 represents the leaves of ZN1‐60117D+ from the disease nursery. IL12 represents the leaves of ZN1‐60127D‐ from the disease nursery. [file TPG2-18-e20557-s008.pdf]

A

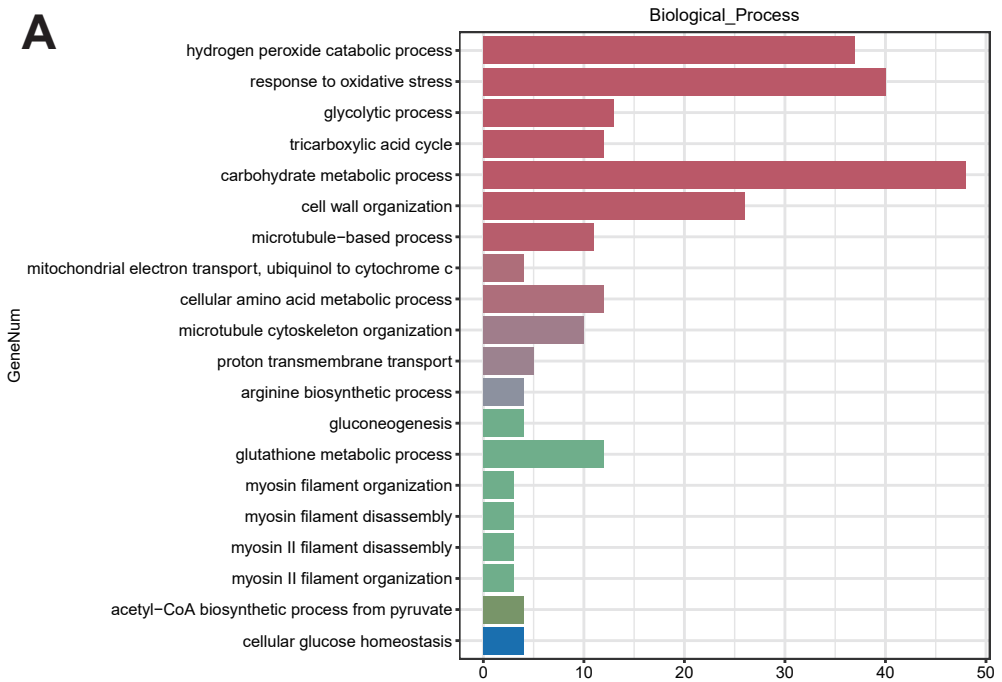

B

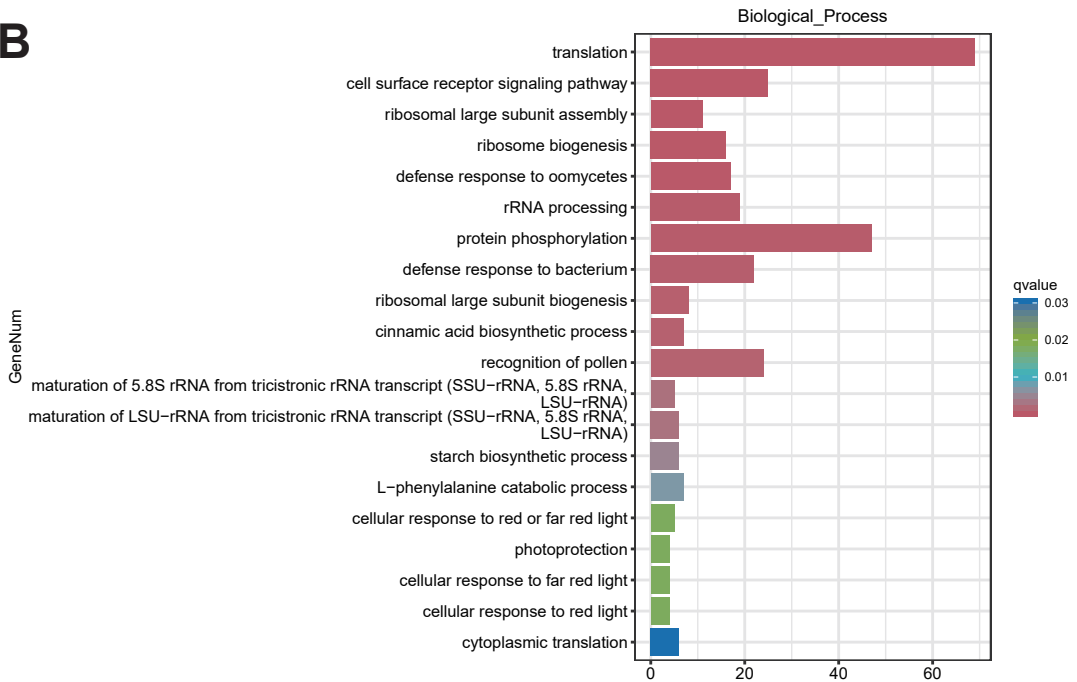

Supplement: Supplementary file 3 — Figure S3. Biological process of GO enrichment. (A) The top 20 significance terms of biological process of differentially expressed genes enriched in roots of near‐isogenic lines. (B) The top 20 significance terms of biological process of differentially expressed genes enriched in leaves of near‐isogenic lines. [file TPG2-18-e20557-s010.pdf]

**A**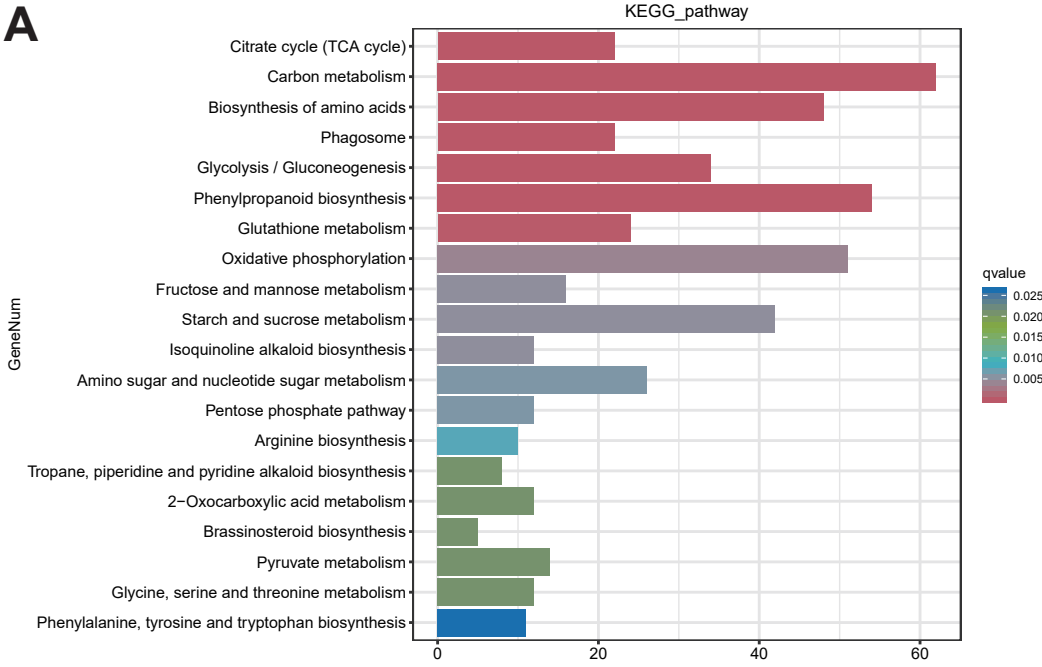**B**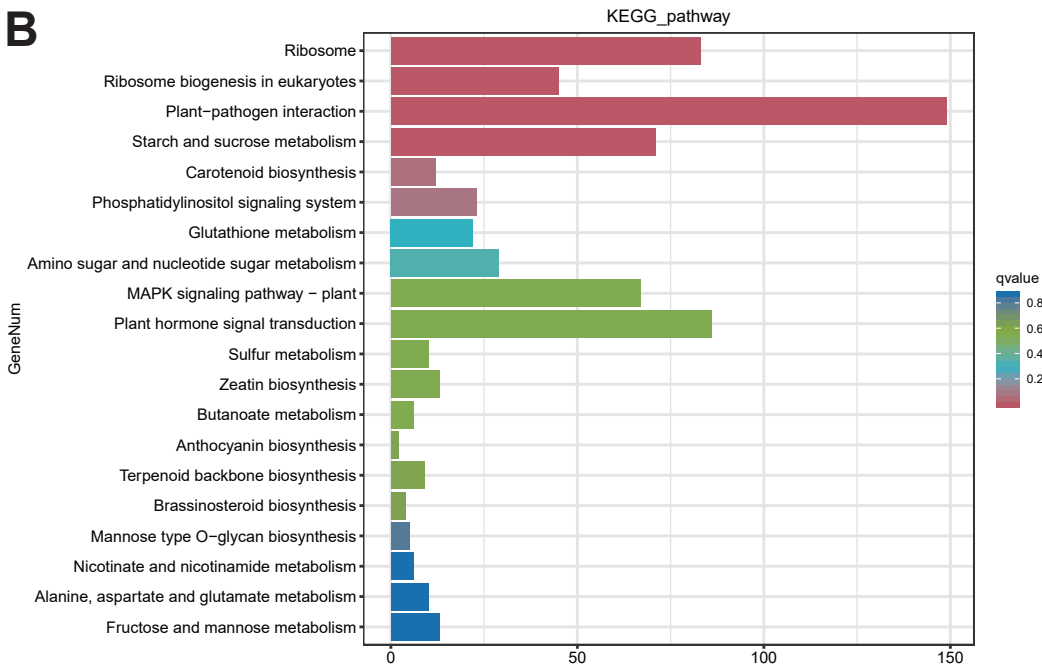

Supplement: Supplementary file 4 — Figure S4. KEGG pathway enrichment. (A) The top 20 significance KEGG pathways of differentially expressed genes enriched in roots of near‐isogenic lines. (B) The top 20 significance KEGG pathways of differentially expressed genes enriched in leaves of near‐isogenic lines. [file TPG2-18-e20557-s007.pdf]
