# Supplementary material for: Identification and characterization of a novel QTL for barley yellow mosaic disease resistance from bulbous barley
Source: Plant Genome. 2025 Jan 13;18(1):e20557. doi: 10.1002/tpg2.20557 (PMC11726411; doi:10.1002/tpg2.20557)
Supplement: Supplementary file 5 — Figure S5. Quantitative real‐time PCR validation of 8 randomly selected differentially expressed genes. Dual‐axis graph comparing gene relative expression level obtained from qRT‐PCR (left y‐axis) and RNA‐seq (right y‐axis). The expression trends of RNA‐seq and qRT‐PCR are consistent, it indicates that the results of RNA‐seq are available. [file TPG2-18-e20557-s002.pdf]

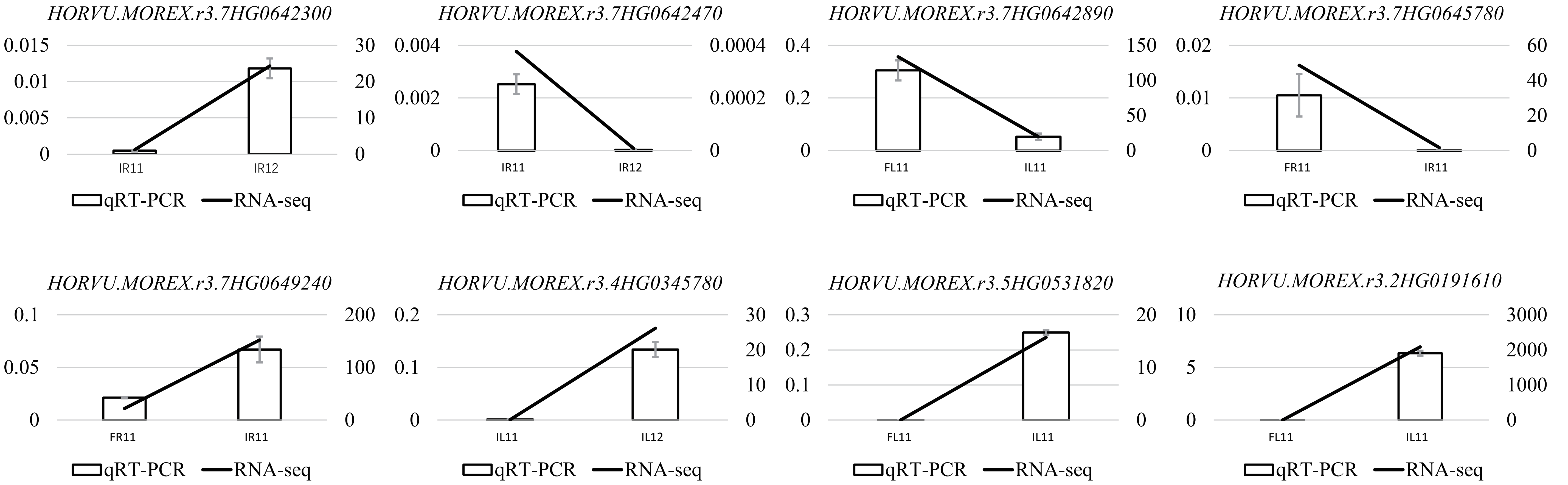
HORVU.MOREX.r3.7HG0642300
0.015
0.01
0.005
0
30
20
10
0
IR11
IR12
qRT-PCR
RNA-seq
HORVU.MOREX.r3.7HG0642470
0.004
0.002
0
0.0004
0.0002
0
IR11
IR12
qRT-PCR
RNA-seq
HORVU.MOREX.r3.7HG0642890
0.4
0.2
0
150
100
50
0
FL11
IL11
qRT-PCR
RNA-seq
HORVU.MOREX.r3.7HG0645780
0.02
0.01
0
60
40
20
0
FR11
IR11
qRT-PCR
RNA-seq
HORVU.MOREX.r3.7HG0649240
0.1
0.05
0
200
100
0
FR11
IR11
qRT-PCR
RNA-seq
HORVU.MOREX.r3.4HG0345780
0.2
0.1
0
30
20
10
0
IL11
IL12
qRT-PCR
RNA-seq
HORVU.MOREX.r3.5HG0531820
0.3
0.2
0.1
0
20
10
0
FL11
IL11
qRT-PCR
RNA-seq
HORVU.MOREX.r3.2HG0191610
10
5
0
3000
2000
1000
0
FL11
IL11
qRT-PCR
RNA-seq
